# Maximization of Markers Linked in Coupling for Tetraploid Potatoes via Monoparental Haploids

**DOI:** 10.3389/fpls.2018.00620

**Published:** 2018-05-07

**Authors:** Annette M. Bartkiewicz, Friederike Chilla, Diro Terefe-Ayana, Jens Lübeck, Josef Strahwald, Eckhard Tacke, Hans-Reinhard Hofferbert, Marcus Linde, Thomas Debener

**Affiliations:** ^1^Department of Molecular Plant Breeding, Institute of Plant Genetics, Leibniz University Hannover, Hannover, Germany; ^2^Westhoff, Südlohn, Germany; ^3^SaKa Pflanzenzucht GmbH & Co. KG, Hohenmocker, Germany; ^4^Böhm-Nordkartoffel Agrarproduktion GmbH & Co. OHG, Ebstorf, Germany

**Keywords:** SNPs, linkage, QTL, mapping, dihaploid, tuber, *Solanum phureja*

## Abstract

Haploid potato populations derived from a single tetraploid donor constitute an efficient strategy to analyze markers segregating from a single donor genotype. Analysis of marker segregation in populations derived from crosses between polysomic tetraploids is complicated by a maximum of eight segregating alleles, multiple dosages of the markers and problems related to linkage analysis of marker segregation in repulsion. Here, we present data on two monoparental haploid populations generated by prickle pollination of two tetraploid cultivars with *Solanum phureja* and genotyped with the 12.8 k SolCAP single nucleotide polymorphism (SNP) array. We show that in a population of monoparental haploids, the number of biallelic SNP markers segregating in linkage to loci from the tetraploid donor genotype is much larger than in putative crosses of this genotype to a diverse selection of 125 tetraploid cultivars. Although this strategy is more laborious than conventional breeding, the generation of haploid progeny for efficient marker analysis is straightforward if morphological markers and flow cytometry are utilized to select true haploid progeny. The level of introgressed fragments from *S. phureja*, the haploid inducer, is very low, supporting its suitability for genetic analysis. Mapping with single-dose markers allowed the analysis of quantitative trait loci (QTL) for four phenotypic traits.

## Introduction

The cultivated potato (*Solanum tuberosum* L.) is a highly heterozygous polysomic tetraploid outcrossing species with 48 chromosomes (2*n* = 4*x* = 48) that shows tetrasomic inheritance and has a haploid genome size of approximately 840 Megabase pairs (Bradshaw, 2007). Progeny from crosses between tetraploid genotypes display complex segregation patterns that severely complicate genetic analyses compared with progeny from diploid parents (Mann et al., 2011).

As a maximum of eight alleles can segregate in progenies from crosses between tetraploid genotypes, marker analysis is also complicated because full resolution of the marker genotypes can only be achieved by the precise determination of allele dosages. Although the latter problem has been solved with recent technological advances in generating single nucleotide polymorphism (SNP) markers (Voorrips et al., 2011; Hackett et al., 2013), detection of linkage between traits and markers that are linked in repulsion remains elusive. Therefore, most of the mapping approaches in tetraploids are based on markers that are linked in coupling.

Most of the genetic studies in potato have been conducted in diploid genotypes (Bonierbale et al., 1988; Gebhardt et al., 1989, 1991; Tanksley et al., 1992), which are often obtained as haploids from tetraploid cultivars by androgenesis or parthenogenesis. An androgenic approach to obtain haploids from a tetraploid cultivar is via anther culture (Uhrig and Salamini, 1987; Rokka et al., 1996), although it has been reported that many tetraploid cultivars do not respond well to this method (Irikura, 1975) and that there is a strong influence of the respective genotype on the success rate (Jacobsen and Sopory, 1978). Song et al. (2005) successfully used 57 primary haploid clones derived from an anther culture for the development of genetic markers for extreme resistance to potato virus Y (*Ry*_*sto*_) in a bulked segregant analysis.

A parthenogenic approach to generate haploids is so-called “prickle pollination” crosses with specific *Solanum phureja* pollinators that induce the development of haploid seed (Hougas and Peloquin, 1957; Hutten et al., 1994). The availability of a dominant seed marker (“embryo spot”) in the haploid inducer genotypes facilitates the removal of undesirable hybrids within the progeny because tetraploid and triploid hybrids of the cross inherit a purple anthocyanin pigmentation at the base of the cotyledons, which is also visible as nodal bands in plant seedlings (Peloquin and Hougas, 1959; Hermsen and Verdenius, 1973). Different cytogenetic mechanisms have been proposed for the haploid formation of crosses with *S. phureja* pollinators. Haploids from such crosses have been regarded as parthenogenetically developed, and it was speculated that *S. phureja* pollen triggers the development of unfertilized egg cells into embryos without making any genetic contribution to the embryo itself (Hermsen and Verdenius, 1973). Clulow et al. (1991) suggested that *S. phureja* chromosomes are eliminated from embryonic cells during cell divisions after the fertilization of egg cells, resulting in haploid progeny. This finding was also reported in later introgression analyses (Clulow and Rousselle-Bourgeois, 1997; Straadt and Rasmussen, 2003; Ercolano et al., 2004). Haploid populations derived by parthenogenesis have been used for genetic analyses (Kotch et al., 1992) and to identify markers that are linked to nematode resistance (Pineda et al., 1993); however, the number of genotypes used in the RFLP mapping approach was very small with 37 haploid individuals.

The first genetic analyses in potatoes were reported as early as 1910 by Salaman, who analyzed the inheritance of male sterility, haulm characteristics, tuber shape and color, as well as eye depth without considering the tetraploidy and tetrasomic inheritance of the cultivated potato. Therefore, genetic analyses were often limited to the inheritance of dominant traits such as the tuber skin color (Black, 1933). The construction of linkage maps was first reported by Bonierbale et al. (1988), who used restriction fragment length polymorphism (RFLP) markers and tomato probes, followed by an RFLP map obtained from diploid *S. tuberosum* clones (Gebhardt et al., 1989, 1991) and high density maps of the tomato and potato genomes (Tanksley et al., 1992). Amplified fragment length polymorphism (AFLP) and simple sequence repeat (SSR) markers have been used extensively in potato research and mapping approaches (Veilleux et al., 1995; Milbourne et al., 1998; Ghislain et al., 2004, 2009; Feingold et al., 2005; van Os et al., 2006). Over the last years, most of the aforementioned markers have been replaced by SNP markers, for which extensive resources are available in potato (Hamilton et al., 2011; Felcher et al., 2012; Uitdewilligen et al., 2013; Vos et al., 2015).

However, traditional potato breeding is carried out at the tetraploid level between tetraploid cultivars, and it is mainly executed by the phenotypic selection of favorable traits (Carputo and Frusciante, 2011). Genetic analysis of traits and trait combinations from particularly interesting tetraploid genotypes is prone to the above mentioned problems. A solution to such problems would be the generation of a larger population of haploids extracted from single elite tetraploids (monoparental haploids). In these populations, 25% of all markers from a particular genomic region should segregate in linkage to one of the four chromatids.

In this study, we present the generation of two large monoparental haploid populations consisting of 215 and 87 individuals, respectively, which were derived from two different tetraploid cultivars. We analyzed the populations and additional unrelated tetraploid cultivars with a large set of SNPs and determined the degree of genome introgression from the *S. phureja* pollinators. The markers were used for mapping of quantitative trait loci in the larger population as well as for analyzing the number of useful single-dose markers in simulated crosses with different tetraploid cultivars.

## Materials and methods

### Plant material

Two tetraploid breeding clones of industrial starch potatoes, P208 and P809, were used for the construction of two haploid populations by so called “prickle pollination” with the haploid inducer clones IVP101 and IVP35 of the diploid wild potato species *Solanum phureja*. Haploid genotypes derived from the cross with P208 were used for SNP genotyping and genetic mapping. A subset of the haploid progeny derived from P809, as well as 125 tetraploid German and Polish cultivars, were also used for SNP genotyping.

### Generation of haploid potato populations

Pollinations with the haploid inducers were performed in the greenhouse on emasculated flowers of P208 and P809. Seeds of the cross of P208 with *S. phureja* IVP35 were preselected for the occurrence of an embryo spot. Seeds derived from the crosses were surface sterilized by incubation for 30 s in 70% ethanol and 2 min in 0.5% sodium hypochlorite + Tween20, followed by three washing steps of 5 min each in sterile distilled water. Seeds were germinated *in vitro* on Murashige Skoog medium (Murashige and Skoog, 1962) containing 3% sucrose and solidified with 8.4 g plant agar (Duchefa Biochemie B.V., Haarlem, The Netherlands) per liter. The emerging seedlings were cultivated at 23°C in a 16 h light/8 h dark cycle with light intensities of approximately 61 μmol m^−2^ s^−1^.

### Ploidy determination

Putative haploid seedlings were visually selected by a lack of anthocyanin pigmentation in the nodes of the *in vitro* seedlings. The ploidy of the selected seedlings was subsequently determined by flow cytometry with a CyFlow Ploidy Analyzer (Partec, Münster, Germany). Leaf tissue (approximately 1 cm^2^) from *in vitro* plantlets was chopped with razor blades in nuclei extraction buffer. Plant nuclei were stained with 4',6-diamidino-2-phenylindole using the CyStain UV Precise P kit (Partec, Münster, Germany). Analyses were performed according to the manufacturer's protocol, counting at least 1,000 nuclei per sample. The parental genotypes with known ploidy were used as standards for diploid and tetraploid genotypes.

### DNA extraction

For DNA extraction, approximately 30 mg of dried leaf tissue was homogenized with a TissueLyser II (Qiagen, Hilden, Germany). DNA was extracted using the DNeasy Plant Mini Kit (Qiagen, Hilden, Germany) according to the manufacturer's protocol. The DNA concentration was determined using a NanoDrop 2000 spectrophotometer (Thermo Fisher Scientific Inc., Waltham, Massachusetts, USA).

### Simple sequence repeat markers

SSR markers were PCR-amplified from 40 ng genomic DNA using primers for markers STI0032, StI031, STI051 (Feingold et al., 2005), STM0031, and STM1052 (Milbourne et al., 1998) for the P208 population and StI047, StI0030 (Feingold et al., 2005), and STM1106 (Milbourne et al., 1998) for the P809 population, to check for introgression of the pollinator genome of *S. phureja*. Forward primers were M13-tailed (5′-GTAAAACGACGGCCAGT-3′) at the 5′-end, and a second M13-forward primer labeled with IRD700 (Eurofins MWG, Ebersberg, Germany) was used (Schuelke, 2000). The PCR mixture consisted of a total volume of 20 μl containing 0.125 μM of the IRD700-labeled M13-forward primer, 0.025 μM of the marker-specific forward primer, 0.25 μM of the marker-specific reverse primer, 1 unit of DCS *Taq* polymerase (DNA Cloning Service e. K., Hamburg, Germany), 1 × Williams buffer (100 mM Tris-HCl (pH 8.0), 500 mM KCl, 20 mM MgCl_2_, 0.01% gelatin) and 0.15 mM of each dNTP. PCR conditions were as follows: initial denaturation for 5 min at 94°C, followed by 25 cycles of 45 s at 94°C, 1 min at 63°C and 1 min at 72°C, eight cycles of 30 s at 94°C, 45 s at 52°C and 1 min at 72°C, and a final extension of 10 min at 72°C. After PCR, 100–250 μl of formamide loading dye (98% formamide, 10 mM EDTA, 0.05% pararosaniline) was added, and samples were denatured for 3 min at 95°C. For each sample, 0.3 μl of diluted PCR product was separated on 6% polyacrylamide gels (Sequagel XR, National Diagnostics, Nottingham, UK) on a LI-COR DNA Analyzer 4300 (LI-COR, Lincoln, Nebraska, USA) according to the manufacturer's protocol.

### Amplified fragment length polymorphism markers

AFLP analysis was performed for the P208 population as previously described by Vos et al. (1995) with minor modifications. For each genotype, 250 ng of DNA was digested with 10 units *Hind*III and 3 units *Mse*I restriction enzymes (New England Biolabs Inc., Ipswich, Massachusetts, USA). Preamplification was performed with adapter-specific primers. Preamplified samples of five or six genotypes were pooled into 42 bulks for the final amplification, for which an IRD700 end-labeled *Hind*III primer (Eurofins MWG, Ebersberg, Germany) with three selective bases (5′-AGACTGCGTACCAGCTT-AAC-3′) and 16 different *Mse*I primers (5′-GACGATGAGTCCTGAGTAA-ANN-3′) with three selective bases (AAA, AAC, AAG, AAT, ACA, ACC, ACG, ACT, AGA, AGC, AGG, AGT, ATA, ATC, ATG, ATT) at the three prime end were used. Fragments were size separated as described above. AFLP analysis was performed subsequently in the individual genotypes of the respective bulks when *S. phureja*-specific marker bands were detected in the bulks.

### SNP genotyping using the 12.8 k SolCAP potato SNP array

Using the 12.8 k SolCAP potato genotyping array, 219 genotypes of the P208 population and 39 genotypes of the P809 population, as well as the parental genotypes and the two pollinator clones of *S. phureja*, were genotyped for 12,808 SNPs (http://solcap.msu.edu/potato_infinium.shtml). P208 and the two *S. phureja* clones were genotyped with two repeats. In addition, 125 tetraploid German and Polish potato cultivars, which are listed in Table S4, were also genotyped. Custom genotyping was performed by Neogene Genomics (Neogene Genomics, Lincoln, Nebraska, USA).

### Kompetitive allele specific PCR assay

SNP array results were validated using Kompetitive allele specific PCR (KASP) markers for the eight SNP markers solcap_snp_c2_10957, solcap_snp_c2_17747, solcap_snp_c2_25560, solcap_snp_c2_32982, solcap_snp_ c2_35942, solcap_snp_c2_41768, solcap_snp_c2_42407 and solcap_snp_c2_52712. KASP primers were designed by LGC Genomics (LGC, Hoddesdon, UK) in a KASP by Design assay based on the context sequence information provided by the Solanaceae Coordinated Agricultural Project (http://solcap.msu.edu/data/potato_69011_map_context_DM_v3_superscaffolds.txt). PCR was performed with 50 ng of genomic DNA in a 10 μl reaction volume on an ABI StepOnePlus instrument (Thermo Fisher Scientific, Waltham, Massachusetts, USA) according to the protocol provided by LGC genomics.

### Genetic mapping

Genetic mapping was performed for the P208 population using only single-dose SNP markers indicated by a 1:1 segregation. Skewed markers or markers with missing values for more than 7% of the genotypes were not considered. Linkage analysis was performed in JoinMap®4 (Van Ooijen, 2006) using the mapping function of Haldane (1919) and the regression mapping algorithm (Stam, 1993). For the construction of the linkage maps LOD scores between 6 and 15 were chosen. For each potato chromosome, two to four linkage maps were constructed.

### Morphological characterization and QTL mapping

For 168 genotypes of the P208 population, three clones per genotype were potted in 5-liter pots with standard potting soil “Einheitserde” (type P) and were phenotyped in the greenhouse under semi-controlled conditions for the four morphological traits: number of tubers, tuber weight, shoot length, and number of nodes. Mean values were calculated for shoot length and number of nodes, which was measured and counted for three clones per genotype. Tubers were harvested from three clones, and the mean value was calculated for the tuber number per plant. All tubers were weighed, and the mean value for weight per tuber was calculated by dividing the overall tuber weight by the overall tuber number for each genotype. Initial testing for a normal distribution of the phenotypes was performed with a Shapiro-Wilk normality test using R software version 3.1.3 (R Development Core Team, 2011). Phenotypic data were Box-Cox transformed (Box and Cox, 1964) using the Free Statistics Software version 1.2.1 (Wessa, 2016) for QTL analysis with MapQTL®6 (Van Ooijen, 2009) with a permutation test with 1,000 permutations and subsequent interval mapping and multiple-QTL mapping with default settings.

## Results

### Generation of the haploid populations and selection of true haploids by morphology and flow cytometry

A subset of seeds from pollinations with the two inducer clones, 1178 seeds for P208 and 498 seeds for P809, were used for *in vitro* germination. Emerging seedlings were preselected morphologically for anthocyanin pigmentation at the nodes of the plantlets. As this pigmentation is inherited from the haploid inducer, it is only visible in triploid and tetraploid progeny. The diploid ploidy level of the morphologically selected seedlings was subsequently confirmed by flow cytometry where genotypes were clearly classified in di-, tri- and tetraploids in relation to the diploid and tetraploid parental genotypes that were used as standards.

For the P208 × *S. phureja* IVP101 cross, we obtained 112 haploid genotypes (11.5%), whereas 106 haploid genotypes (51.5%) were generated from the P208 × *S. phureja* IVP35 cross, where seeds were pre-selected for an embryo spot before germination (Table 1). Altogether, 218 haploid genotypes for P208 were generated. The P809 × *S. phureja* IVP101 cross resulted in 89 haploid genotypes (17.9%). Non-germinated seeds were not taken into consideration for the percentages.

**Table 1:** Establishment of two monoparental haploid populations derived from two different tetraploid cultivars.

**Cross**	**Number of seeds used for *in vitro* germination**	**Number of haploid genotypes**	**Percentage of haploid genotypes**
P208 × *S. phureja* IVP101	972	112	11.5%
P208 × *S. phureja* IVP35	206	106	51.5%
P809 × *S. phureja* IVP101	498	89	17.9%

### Test of introgression of *Solanum phureja* DNA into the haploid potato populations

**Figure 1:**
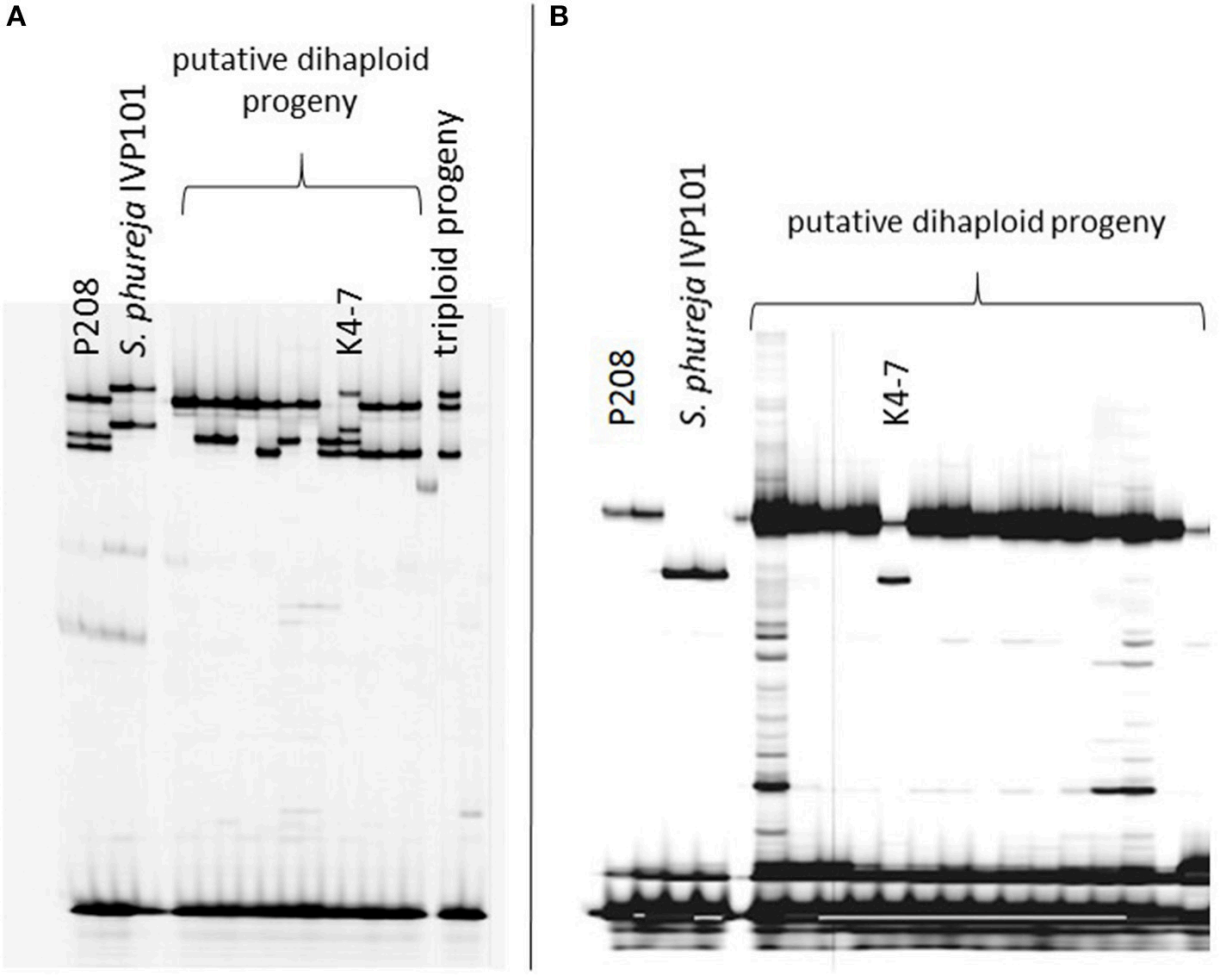
Polyacrylamide gel electrophoresis of SSR markers StI031 **(A)** and STI051 **(B)** analyzed in a subset of putative haploid genotypes of the P208 × *S. phureja* IVP101 cross. Genotype K4-7 showed a *S. phureja*-specific allele for both SSR markers in addition to the maternal alleles.

**Figure 2:**
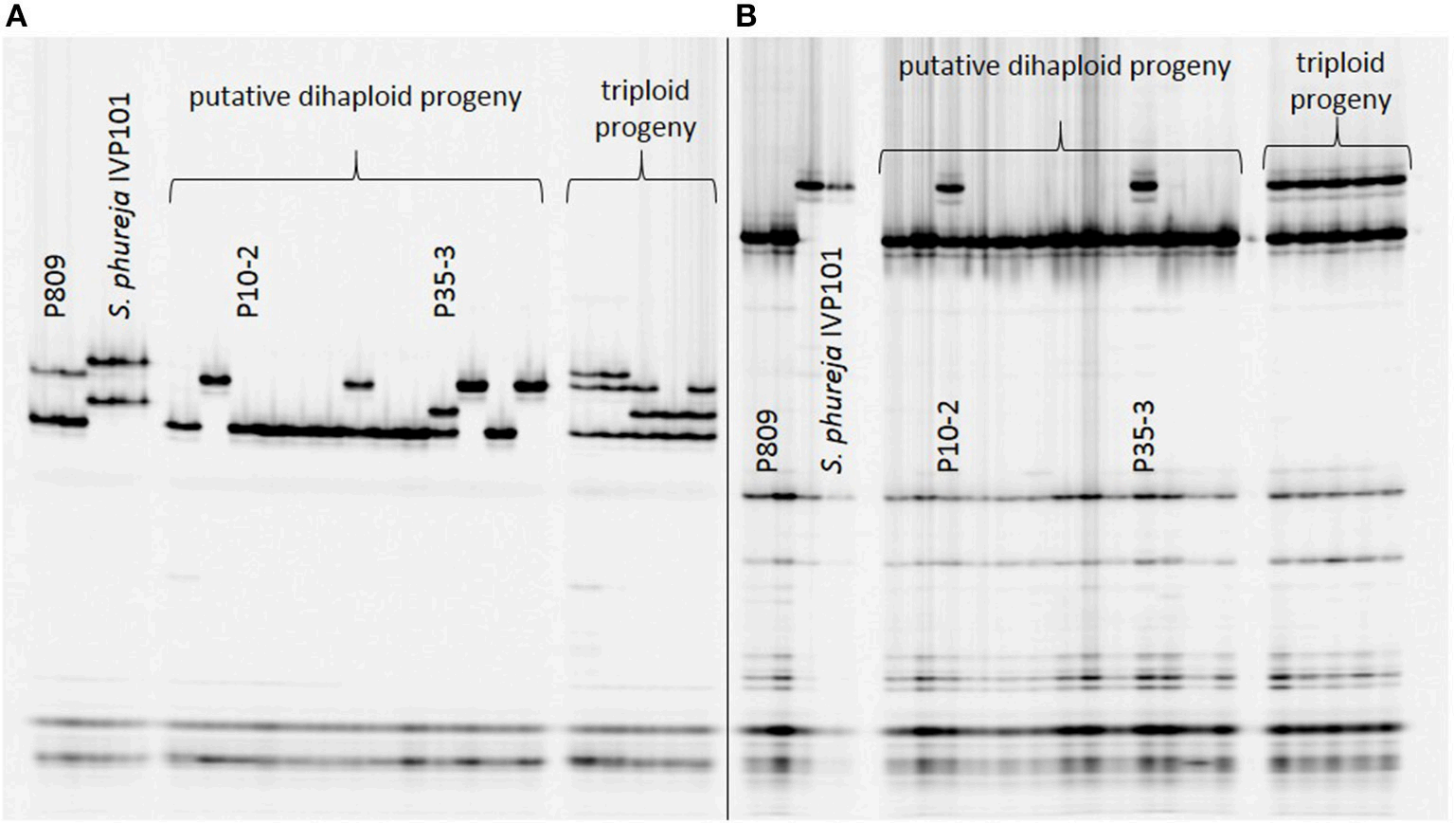
Polyacrylamide gel electrophoresis of SSR markers StI047 **(A)** and StI0030 **(B)** analyzed in a subset of putative haploid genotypes of the P809 × *S. phureja* IVP101 cross. Genotype P35-3 showed a *S. phureja*-specific marker band for SSR markers StI047 and StI0030. With SSR marker StI0030, genotype P10-2 was additionally identified to show a *S. phureja* introgression.

Introgression analysis was performed using different SSR markers that showed fragments specific for the male haploid inducer. The 218 haploid genotypes derived from P208 were screened with five SSR markers: STI0032, StI031, STI051, STM0031, and STM1052. Genotype K4-7 showed at least one *S. phureja*-specific marker band for the markers (Figure 1). The 89 haploid genotypes of P809 were screened with three SSR markers: StI0030, STI047 and STM1106. With SSR marker StI0030, genotype P35-3 showed a putative introgression of *S. phureja* DNA. SSR marker StI047 additionally identified a second genotype, P10-2, with a putative introgression (Figure 2), whereas with the SSR marker STM1106, none of the genotypes showed a *S. phureja*-specific marker band. AFLP analysis with bulks of five to six genotypes of the P208 population was performed and revealed one additional genotype, B35F-11, which showed three *S. phureja* specific marker bands for the primer combination *Hind*III-AAC/*Mse*I-ACT (data not shown).

All 218 genotypes of the P208 population and a subset of 39 genotypes of the P809 population, as well as the parental genotypes and the two pollinator clones *S. phureja* IVP101 and IVP35, were genotyped for 12,808 SNPs using the SolCAP potato genotyping array. The SNP marker data were used to check for additional *S. phureja* introgressions in the haploid genotypes. Genotypes K4-7 and B35F-11 were excluded from further analysis because they showed putative introgressions in the SSR and AFLP analyses, as well as one additional genotype that did not allow proper SNP genotyping. For the P208 × *S. phureja* IVP101 cross, 647 SNP markers were analyzed that were homozygous for one allele in P208 and heterozygous or homozygous for the other allele in the pollinator. For the P208 × *S. phureja* IVP35 cross, 633 SNP markers were suitable for introgression analysis, and for the P809 × *S. phureja* IVP101 cross, 795 SNP markers showed according allele configurations. Introgression analysis revealed 12 SNPs for each of the P208 × *S. phureja* IVP101 and P208 × *S. phureja* IVP35 crosses, and 27 SNPs for the P809 × *S. phureja* IVP101 cross, indicating a putative introgression of the pollinator DNA of *S. phureja* into the haploid progeny (Table S1).

The percentages of haploid genotypes that showed a putative *S. phureja* introgression for the individual SNP markers ranged from 0.9 to 57.66% for the P208 × *S. phureja* IVP101 cross, from 0.94 to 66.04% for the P208 × *S. phureja* IVP35 cross and from 2.56 to 69.23% for the P809 × *S. phureja* IVP101 cross (Table S1). *S. phureja*-specific markers could be detected on all 12 chromosomes, excluding chromosomes 1 and 10 in the haploid progeny derived from P809. SNP markers solcap_snp_c2_54921 and solcap_snp_c2_52621 could not be located on any potato chromosome in the potato genome browser.

In the two populations derived from P208, introgression rarely occurred with more than one marker per chromosome. Introgressions on the same chromosome showed only a few overlapping genotypes. In the P809 population, introgression markers were more commonly located on the same chromosomes. Most notably, two marker pairs of chromosomes 4 (solcap_snp_c2_26773 and solcap_snp_c1_3311) and 8 (solcap_snp_c2_29491 and solcap_snp_c1_6140) showed large numbers of overlapping genotypes, with 18 of 19 and 22 of 24 genotypes, respectively, showing introgression of the pollinator genome (Table S2).

Nearly all haploid genotypes displayed single SNP markers that were specific for *S. phureja*. Only two genotypes, one for each of the crosses with P208, did not show any *S. phureja*-specific allele configuration in the SNP genotyping. The percentages of *S. phureja* markers that putatively introgressed into the individual haploid genotypes were very low, ranging from 0.16 and 1.24% for the P208 × *S. phureja* IVP101 cross and from 0.16 to 1.26% for the individual haploids from the P208 × *S. phureja* IVP35 cross, with an average of 0.53 and 0.56%, respectively (Table S3). For the P809 × *S. phureja* IVP101 cross, all genotyped individuals of the haploid progeny showed an introgression of the *S. phureja* genome ranging from 0.63 to 1.89%, with an average of 1.34% (Table S3).

To validate these results, a KASP assay was performed in the P208 population for eight introgression markers. For three markers, separation of the different SNP genotypes was not possible in the cluster plots. KASP assays were consistent with the SNP array genotyping for three markers in the haploid genotypes. The two remaining markers (solcap_snp_c2_32982 and solcap_snp_c2_42407) showed different results in the KASP assay and on the SNP array for one or two genotypes, respectively (Table 2), indicating calling errors.

**Table 2:** Comparison of SNP genotyping results between the SolCAP SNP array and the KASP assay in the P208 population.

**SNP marker**	**Consistent results**	**Differing results**	**NA SNP array**	**NA KASP assay**
solcap_snp_c2_17747	96.35%	0%	1.37%	2.28%
solcap_snp_c2_25560	97.26%	0%	0.91%	1.83%
solcap_snp_c2_32982	98.17%	0.46%	0.46%	0.46%
solcap_snp_c2_35942	91.78%	0%	7.76%	0.46%
solcap_snp_c2_42407	92.24%	0.91%	6.39%	0.46%

*Percentages of not available (NA) marker data for both methods are also listed*.

### Genetic mapping in a haploid potato population

SNP marker data obtained from genotyping with the 12.8 k SolCAP SNP array was used for genetic mapping in the haploid P208 population. The SNPs were quality filtered, and only markers with less than 15 missing values within the haploid potato population were considered for mapping. Of these 9,953 markers, 9,286 markers (93.3%) showed identical SNP calling results in two repetitions for P208. Altogether, 647 SNP markers showed missing values in at least one repetition in P208, and different genotyping results were identified for 20 SNP markers (0.2%) in the two repetitions. These markers were excluded from further analyses. Of the 9,286 SNP markers that showed identical results regarding allele configurations in the two repetitions of P208, 4,682 SNP markers (50.4%) segregated in the haploid P208 population (Table 3). For construction of the genetic maps, 2,548 single-dose SNP markers displaying a 1:1 segregation were used.

**Table 3:** Summary of the SNP marker data derived from the SNP array in the haploid potato population derived from P208.

**Filtering criteria**	**No. of SNPs**	**Segregation ratios**	**No. of SNPs**
All SNPs	12,808		
After quality filtering	10,376		
Less than 15 missing values	9,953		
Identical in both samples of P208	9,286		
Segregating in the DH population	4,682		
Allele configuration AA:AB	1,358	1:1 segregation	1,129
		5:1 segregation	32
		Distorted segregation	197
Allele configuration BB:AB	1,701	1:1 segregation	1,419
		5:1 segregation	32
		Distorted	250
Allele configuration AA:AB:BB	1,573		
Allele configuration AA:BB	51		

**Figure 3:**
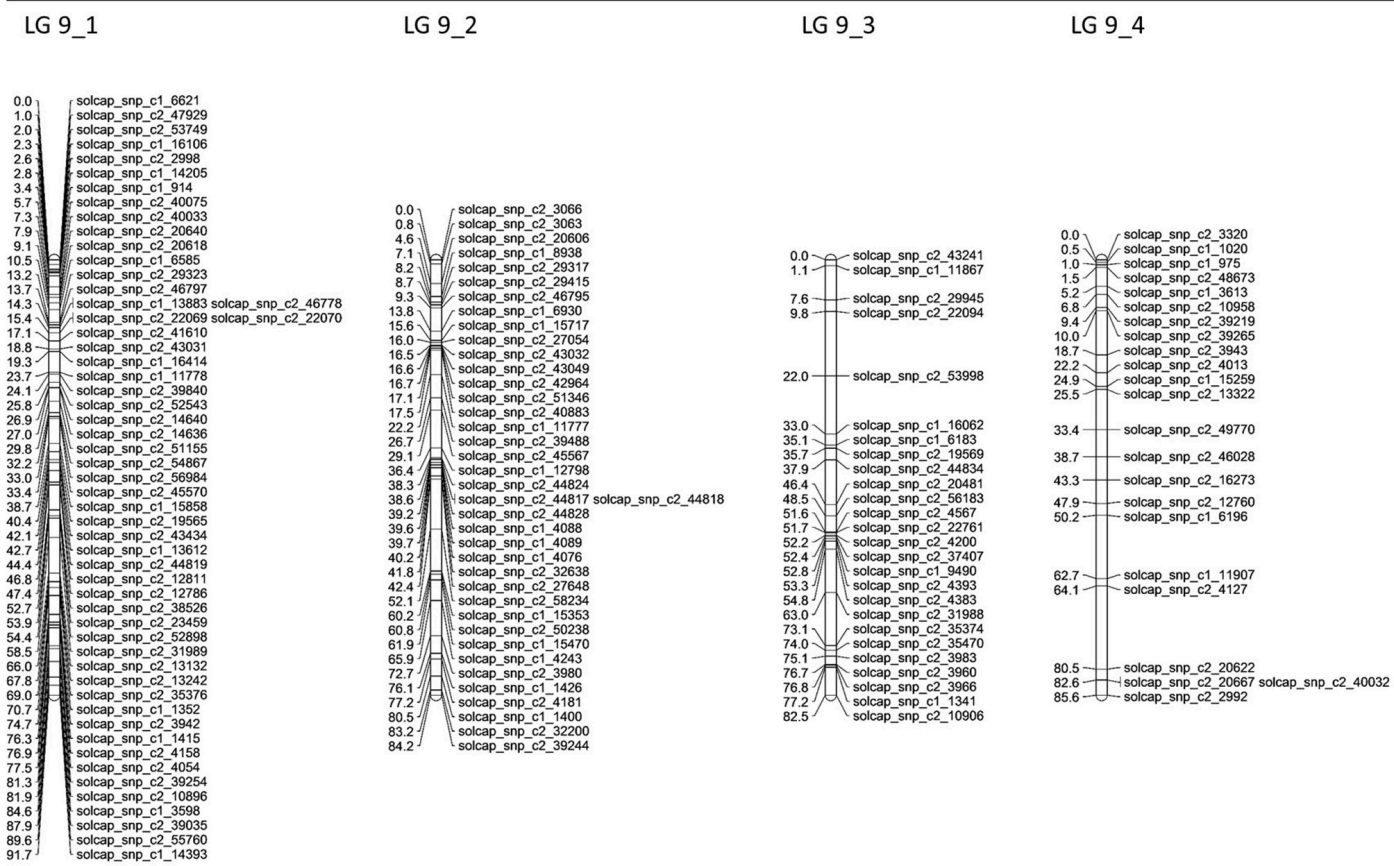
Linkage groups constructed in JoinMap4 with single-dose SNP markers segregating in the P208 population. The four linkage groups represent potato chromosome 9. In total, 268 SNP markers could be mapped for this chromosome, with an average of 67 SNP markers per linkage group. SNP markers showing the same segregation pattern in the population as previously mapped markers were excluded during the mapping process for reasons of clarity.

Altogether, 45 linkage groups were constructed, with LOD scores between 6 and 15, with a total of 2,387 mapped SNP markers, from which 1,290 markers were excluded during the mapping process to provide a better overview because they showed an identical segregation pattern to previously mapped markers. One hundred and sixty-two markers could not be mapped on any linkage group or were excluded manually. For each of the potato chromosomes, four linkage groups were constructed, excluding chromosomes 2 and 6, where only three and two linkage groups could be constructed, respectively (Table 4). The total length of the genetic maps was 2,675.6 cM, with an average of 55.24 SNP markers per linkage group. The genetic maps for the four linkage groups of potato chromosome 9 are shown in Figure 3. Genetic maps for all 12 potato chromosomes are shown in Figures S1–S12.

**Table 4:** Number of linkage groups per chromosome that were constructed for the P208 population with the average number of SNP markers per linkage group.

**Chromosome**	**No. of LGs**	**Average no. of loci per LG**
1	4	61.75
2	3	64.67
3	4	41.25
4	4	55.25
5	4	55.25
6	2	100.5
7	4	55.25
8	4	37
9	4	67
10	4	46.25
11	4	30.25
12	4	48.5

To evaluate whether a monoparental population allows the mapping of more markers in linkage from the donor genotype compared to a conventional cross with a second tetraploid parent, we analyzed theoretical numbers of putatively segregating single-dose markers in conventional crosses between P208 and a second tetraploid parent. Using the SNP marker data available from the SNP array for 125 tetraploid cultivars of different breeding origins (http://www.bdp-online.de/de/GFPi/Abteilungen_Projekte/Abteilung_Kartoffeln/EU_Projekt__Cornet-SynTest/Participating_Breeders/), all 2,548 single-dose markers identified for P208 were considered, and the tetraploid cultivars were screened for markers with one or more doses of the same marker allele. In a cross with such a parent, those markers would no longer segregate as single-dose markers but would display more complex segregation patterns. The number of useful single-dose markers in a tetraploid cross segregating in linkage to loci on one of the four chromatids of P208 would be reduced to 543–928 SNP markers, representing 21.31–36.42% of the useful SNPs in our haploid population (Table 5). Detailed SNP marker information for each tetraploid cultivar is provided in Table S4.

**Table 5:** Number of single-dose markers derived from P208 in putative progenies from crosses to various tetraploid cultivars.

**Number of single-dose markers in a cross between P208 and a second tetraploid cultivar**	**Number of tetraploid cultivars**	**Percentage of useful SNP markers compared to single-dose markers in P208**
<600	2	<23.55%
601–700	23	23.59–27.47%
701–800	66	27.51–31.40%
801–900	32	31.46–35.32%
>900	2	>35.36%

*The numbers of putative single-dose markers in a biparental cross for 125 tetraploid cultivars are listed, as well as the percentage of useful single-dose markers in a biparental cross when compared to the 2,548 single-dose markers in the P208 population*.

### Morphological characterization and QTL mapping

For 168 genotypes of the P208 population, three clones per genotype were phenotyped for shoot length, number of nodes, number of tubers and tuber weight. For the remaining genotypes of the P208 population, acclimatization from *in vitro* culture was not successful, or the overall plant vigor was very low, and therefore they were not included in the analysis.

The average shoot length ranged from 2 cm to 167.33 cm, and the mean number of nodes was between 3 and 36.3. The average tuber number ranged from 0 to 101.67 tubers per plant, with a mean weight per tuber between 0.23 and 200.29 g (Figures S13–S16).

Only the data for the average number of nodes showed a normal distribution, with a *p*-value of 0.1726 based on the Shapiro-Wilk normality test (Table S5). The phenotypic data for the remaining traits was transformed using a Box-Cox transformation with subsequent testing for a normal distribution. Box-Cox-transformed data for the average number of tubers and average tuber weight showed a normal distribution with a *p*-value of 0.3171 and 0.3685, respectively. A normal distribution of the average shoot length was assumed for the QTL analyses. Box-Cox-transformed data for all phenotypic traits showed significant positive correlations (Table S6).

**Figure 4:**
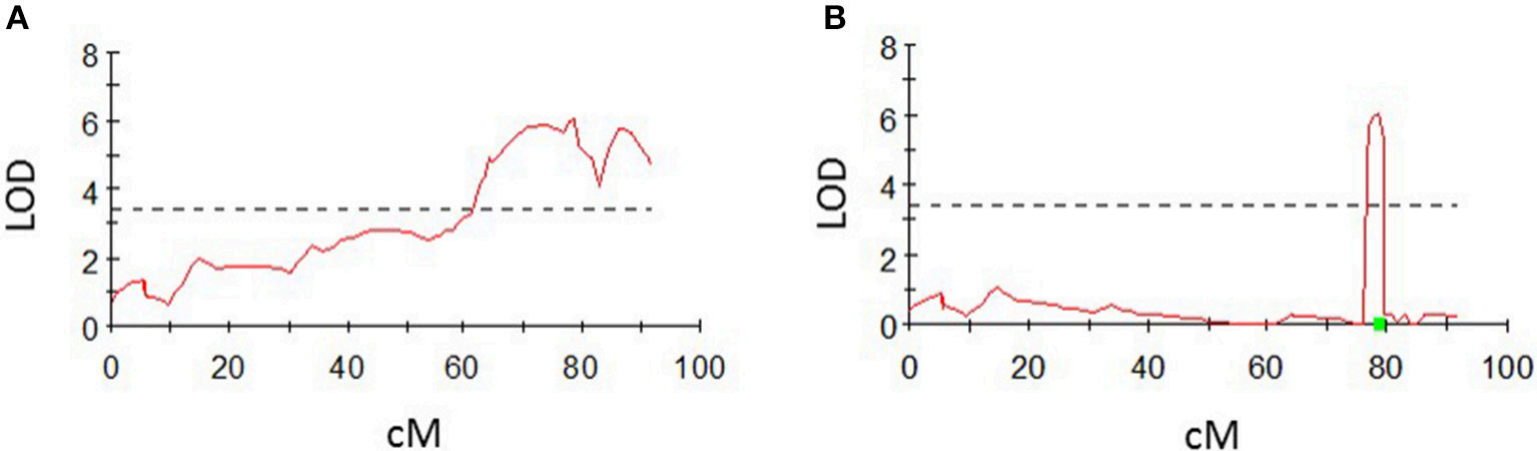
LOD profile on a linkage group for chromosome 4 for the average number of tubers using interval mapping **(A)** and multiple-QTL mapping **(B)**. LOD values above the significance threshold of 3.4 (dashed line) were detected with both approaches. The QTL intervals could be narrowed down from approximately 30 cM to less than 3 cM using the multiple-QTL mapping approach.

For the QTL analyses, a genome-wide LOD score of 3.4 was chosen after permutation tests with 1,000 permutations for all phenotypic traits in MapQTL®6, except for the average number of nodes, for which a genome-wide LOD score of 3.2 was chosen. Significant QTL were found for all phenotypic traits by interval mapping (Table 6). Two QTLs for shoot length were detected on potato chromosomes 2 and 4, two QTLs for the number of nodes were found on chromosomes 4 and 5 and one significant QTL each was detected for the tuber number and tuber weight on potato chromosome 4. No additional QTLs were detected using the multiple-QTL mapping approach, but the QTL for shoot length located on chromosome 2 could not be detected using this approach. Intervals could be minimized in the multiple-QTL mapping approach in comparison to the interval mapping, except for the QTL on chromosome 4 for the number of nodes. The QTL interval for tuber numbers located on chromosome 4, for example, could be minimized from approximately 30 cM in the interval mapping to less than 3 cM in the multiple-QTL mapping approach (Figure 4). QTL charts for all phenotypic traits are shown in Figures S17–S22.

**Table 6:** Quantitative trait loci (QTL) in respective chromosome (chr.) and linkage group (LG) for tuber weight (TW), number of tubers (TN), shoot length (SL) and number of nodes (N) using an Interval mapping (IM), and a multiple-QTL mapping approach (MQM).

**Trait**	**Mapping approach**	**Chr**.	**LG**	**Position [cM]**	**LOD score**	**Marker with highest LOD score**	**Physical position**	**Position [cM]**	**LOD score**	**Expl. variance [%]**
TW	IM	4	4_1	56.758–91.810	3.65–12.92	solcap_snp_c2_29872	2,714,003	86.160	12.92	28.5
TW	MQM	4	4_1	82.861–91.810	9.73–12.92					
TN	IM	4	4_1	62.067–91.810	3.79–6.07	solcap_snp_c2_11569	6,210,370	78.504	6.07	14.5
TN	MQM	4	4_1	76.792–79.504	5.31–6.07					
SL	IM	2	2_1	41.238–42.917	3.72–3.77	solcap_snp_c1_13236	31,663,265	42.917	3.73	9.7
				44.612–57.206	3.61–4.48	solcap_snp_c2_38952 /solcap_snp_c2_52135	28,230,202/ 28,112,384	51.912/51.925	4.48	11.6
				58.994–61.852	3.41–3.52	solcap_snp_c2_38007	21,868,087	61.852	3.52	9.2
SL	MQM	2	2_1	–	–	–		–	–	–
SL	IM	4	4_1	75.870–91.810	3.47–6.74	solcap_snp_c2_29872	2,714,003	86.160	6.74	16.9
SL	MQM	4	4_1	82.861–85.861	3.91–5.78	solcap_snp_c2_45927	3,562,152	82.861	3.91	9.0
N	IM	4	4_1	84.861–91.810	3.35–4.05	solcap_snp_c2_29872	2,714,003	86.160	4.00	10.4
N	MQM	4	4_1	83.861–91.810	3.20–4.25				4.25	10.0
N	IM	5	5_4	97.000–110.467	3.32–3.70	solcap_snp_c2_50317	NA	108.824	3.57	9.3
N	MQM	5	5_4	96.000–108.824	3.65–4.06				3.81	8.9

*The positions of the respective QTL are listed, as well as the SNP (Single nucleotid polymorphism) markers with the highest LOD (Logarith of odds) score and the explained variance*.

## Discussion

Although, haploid techniques have been used for decades to improve cultivar breeding in potato (Hougas and Peloquin, 1958; Hougas et al., 1958; Chase, 1963), conventional breeding programs are almost exclusively conducted at the tetraploid level. In contrast, genetic studies have mostly been carried out at the diploid level with haploid genotypes crossed to diploid potato species. Genetic analyses in which monoparental haploid clones have been used directly are very rare, and the population sizes were very small with only 37 (Pineda et al., 1993) and 57 haploid individuals (Song et al., 2005). In this study, we show that the generation of monoparental haploid populations is a useful tool to dissect interesting traits of an polysomic tetraploid genotype for genetic analyses. To our knowledge, this is the first study to show the direct use of a large population of monoparental haploids for the construction of genetic linkage maps and QTL mapping.

The procedure we chose to select haploid progeny using a combination of morphological markers and flow cytometry, although more laborious than conventional tetraploid crosses, is an efficient strategy for the generation of such populations because most of the unwanted triploid and tetraploid progeny can be easily identified phenotypically by the presence of anthocyanin pigmentation in the nodes. As flow cytometry allows the rapid high-throughput screening of large numbers of samples, the selection of haploid genotypes is a straightforward approach. The frequencies of haploids obtained from *S. phureja* IVP101 crosses were relatively low at 11.5 and 17.9%, but considerably higher after pre-selection for an embryo spot in the cross of *S. phureja* IVP35 with 51.5% haploid genotypes (Table 1). Hutten et al. (1994) showed that when comparing the haploid induction ability of different haploid inducers of *S. phureja*, clone IVP101 performed better than clones IVP35 and IVP48, indicating that pre-selection for an embryo spot on the seeds before *in vitro* germination is an efficient way to increase the number of haploid genotypes. However, pre-selection for an embryo spot on the seeds was found to be very laborious and time-consuming due to the dark seed color of seeds derived from P208. Pre-selection for anthocyanin pigmentation in the nodal bands of the seedlings, however, was very efficient for selecting true haploids, and diploid ploidy was confirmed by flow cytometry (data not shown).

It has been reported that haploid induction via anther culture may be preferable to *S. phureja* pollination with higher frequencies of haploids and faster shoot formation (Schwarzfischer et al., 2002), although both haploid techniques are laborious and time-consuming. One major disadvantage that has been reported in haploids derived from *S. phureja* pollinations is the introgression of the pollinator genome in the haploid progeny (Clulow et al., 1991). Our results showed that one and two haploid genotypes of the P208 population and the P809 population, respectively, contained significant introgressions, indicating that elimination of *S. phureja* chromosomes occurs after fertilization and may be genotype-specific for the tetraploid parental genotype.

Genotyping with the 12.8 k SNP array revealed additional putative *S. phureja* introgressions in the haploid progeny. The number of SNP markers that could be used for introgression analysis was relatively low as only 633–795 SNP markers could be considered that were homozygous for one allele in the tetraploid parental genotypes and heterozygous or homozygous for the second allele in the pollinator genotype. SNP markers specific for the *S. phureja* inducers occurred in almost all genotypes and on nearly all of the potato chromosomes (Table S1). These results are in contrast to those of Straadt and Rasmussen (2003), who observed no introgression of pollinator DNA in 30 haploid genotypes derived from crosses with *S. phureja* IVP101 using AFLP markers for their introgression analysis but also indicated that the introgression rate may be influenced by the tetraploid *S. tuberosum* seed parent. Rarely, more than a single introgression marker could be detected on the same chromosomes in the P208 population, but more often in the P809 population. Markers indicating introgression located on the same chromosome were further analyzed to distinguish true introgression events from putative artifacts due to genotyping errors. Therefore, adjacent markers on either side of the introgression markers were evaluated according to Bourke et al. (2015), who analyzed the occurrence of double reduction rates in tetraploids and used a strict criterion to distinguish true double reduction events from genotyping errors. They assumed a double reduction only when three consecutive markers showed the expected allele configurations. When applying this criterion in our populations, none of the markers would fulfill the requirements for a true introgression. However, the marker density was relatively low, and sometimes the distances between two markers were quite large or the markers showing an introgression did not have adjacent markers with differential allele configurations in the parental genotypes on both sides. An exception to this phenomenon are the markers solcap_snp_c2_29491 and solcap_snp_c1_6140 on chromosome 8, which are located proximately to one another and show highly consistent occurrences in the same genotypes. These markers could indicate a true introgression of the pollinator genome, although they do not fulfill the criterion used by Bourke et al. (2015). Therefore, our results indicate that the overall percentages of the *S. phureja* genome in the haploid populations are very low and do not disturb genetic analyses at the diploid level. This result is supported by verification of selected SNP array marker data for the P208 population using a KASP assay. KASP markers have been used in various plant species for SNP genotyping and validation (Cortés et al., 2011; Rosso et al., 2011; Byers et al., 2012) and for the determination of allele dosages in polyploids (Cuenca et al., 2013). Of the five SNP markers that could be analyzed in the population, two markers, solcap_snp_c2_32982 and solcap_snp_c2_42407, showed differing results in the KASP assay for one or two genotypes, respectively (Table 2).

Genetic mapping was performed in the P208 population using single-dose marker data obtained from the 12.8 k SNP array. Our approach did not include multidose markers, an approach used by many researchers to improve the map density (da Silva et al., 1995; Kriegner et al., 2003; Aitken et al., 2007; Hackett et al., 2013), because herein we focused only on markers segregating in linkage from the tetraploid donor genotype. Furthermore, single-dose markers allow higher mapping precision, and with a population size of 218 progeny from which only 168 could be phenotyped, map resolution was considered to be sufficient using only single-dose markers. The fraction of markers that displayed distorted segregation was 16.7% of all segregating simplex markers and is within the range of other observations in diploid potatoes where 6–57% of markers showed distorted segregation (Gebhardt et al., 1991; Felcher et al., 2012; Manrique-Carpintero et al., 2016). With a total of 45 linkage groups that were constructed with 2,387 mapped markers (93.7% of the simplex markers), only two of the 12 potato chromosomes were represented by less than four linkage groups, which could either be due to large homozygous stretches on these chromosomes or because insufficient numbers of markers were mapped. However, the number of mapped markers in this study is considerably higher compared to linkage maps that were constructed using mostly AFLP and SSR markers for tetraploid (Meyer et al., 1998; Bradshaw et al., 2004, 2008; Bryan et al., 2004) or diploid potato populations (Gebhardt et al., 1989, 1991; Bryan et al., 2002), with only a few hundred mapped markers. Notable exceptions are the high-density map constructed by van Os et al. (2006) with more than 10,000 AFLP markers and the tetraploid maps constructed by Hackett et al. (2013) with 3,839 mapped SNP markers.

We attempted to evaluate whether a monoparental population allows the mapping of more markers in linkage from the donor genotype compared to a conventional cross with a second tetraploid parent in which the heterozygosity for the same marker alleles present in the donor genotype leads to more complex banding patterns. Therefore, we analyzed the theoretical number of segregating single-dose markers of P208 crossed with different tetraploid genotypes for which SNP information from the SNP array was available. In summary, we found that a significant number of markers currently segregating as single-dose markers in our haploid population had a match with at least one dose in the putative parents (Table 5; Table S4), which would reduce the number of useful single-dose markers of P208 to only 21.31% (543) up to 36.42% (962) of SNP markers from the 2,548 simplex SNP markers segregating in the monoparental haploid population. Thus, a much larger fraction of single-dose markers from the maternal parent can be analyzed in our monoparental population because no markers from a second parent will complicate marker segregation patterns. Additionally, all additional single-dose markers from a second tetraploid parent will automatically segregate in repulsion and therefore are only of limited use for mapping maternal traits in conventional crosses. Although gamete formation on the maternal side is not different from a conventional tetraploid cross, segregation patterns are much simpler because only combinations of two alleles are present. While this is not different from the frequently used populations from two haploids, only maternal alleles segregate. Alternative approaches to analyzing the genetics of the tetraploid donor are less effective and more time and resource-consuming. Intercrosses of single haploids would require an additional generation and would suffer from self-incompatibility that renders a number of cross combinations unsuccessful. Furthermore, to capture the whole genetic variation of a tetraploid, a large number of successful cross combinations would be needed.

To demonstrate the additional utility of our monoparental haploid population, QTL were analyzed for four phenotypic traits: number of tubers per plant, tuber weight, shoot length and number of nodes using an interval and a multiple-QTL mapping approach. Many QTL studies have been conducted in potato for various agronomic traits and yield characteristics (Bonierbale et al., 1993; Freyre and Douches, 1994; Schäfer-Pregl et al., 1998; Bradshaw et al., 2008; McCord et al., 2011), as well as quantitative resistances to late blight (Leonards-Schippers et al., 1994; Collins et al., 1999; Danan et al., 2011; Massa et al., 2015) and to cyst nematodes (Rouppe van der Voort et al., 1998; Bryan et al., 2002). More recently, Schönhals et al. (2017) mapped QTLs for tuber yield, starch content and starch yield in three populations of tetraploid cultivars and breeding clones and identified genomic regions on all 12 chromosomes with QTL for the analyzed traits. Rak et al. (2017) identified multiple QTL for several tuber traits in a biparental potato population and found three QTL for the number of tubers on chromosomes 4, 5 and 10 and two QTL for tuber weight, width and length on chromosomes 5 and 6. In this study, we identified one QTL each for tuber number and tuber weight on chromosome 4, explaining 14.5 and 28.5% of the variance of these phenotypic traits (Table 6). QTL for both traits are located closely to one another, where the markers with the highest LOD score span a genomic region of less than 3.5 Mbp, indicating that the underlying genes for tuber number and tuber weight are tightly linked. This locus on the short arm of chromosome 4 represents an additional QTL for tuber number to that of Rak et al. (2017), which is located approximately 60 Mbp farther on the long arm of the same chromosome. Additional QTL were identified for shoot length on chromosomes 2 and 4 and for number of nodes on chromosomes 4 and 5. In the interval mapping, the marker with the highest LOD score was the same for both traits, explaining 16.9% of the variance in shoot length and 10.4% of the variance in the number of nodes (Table 6). Additionally, shoot length and number of nodes were highly correlated with a correlation coefficient of 0.91 (Table S5), indicating that these traits are controlled by the same underlying gene on chromosome 4 but are additionally controlled by different genes located on chromosomes 2 and 5 for shoot length and number of nodes, respectively. A QTL for plant height on chromosome 5 has also been described by Bradshaw et al. (2004) and Hackett et al. (2014) and is located in the same chromosomal region as the QTL we found for the number of nodes. These results show that QTL mapping in a monoparental haploid population is suitable to confirm known as well as to detect additional QTL in a single tetraploid cultivar. Nevertheless, QTL mapping is highly dependent on the genetic background of the studied populations and must be analyzed in a tetraploid genetic background for applications in commercial breeding.

## Conclusions

This is the first study, to our knowledge, to present the direct use of a large monoparental haploid potato population consisting of more than 200 genotypes from a single tetraploid parent for genetic analyses and molecular mapping. Introgression of the *S. phureja* pollinator genome was very low within the progeny. Construction of genetic linkage maps using single-dose SNP markers as well as QTL mapping of four phenotypic traits was successful, with a considerably higher number of SNP markers segregating in linkage than in conventional crosses between two tetraploid genotypes. Although the construction of haploid populations is more laborious than in conventional breeding, our approach represents a promising strategy with monoparental haploids as a useful tool for genetic analysis of a single tetraploid potato cultivar circumventing problems associated with tetraploid genetics.

## Author contributions

AB: Performed the experiments, analyzed the data, prepared all tables and figures and wrote the manuscript; FC: Generated the populations and determined the ploidy of genotypes by flow cytometry: DT-A: Planned and supervised the experiments; JL, JS, ET, and H-RH: Provided the plant material for the crossings and planned the experiments; ML: Planned and supervised the experiments and corrected the manuscript; TD: Planned and supervised the experiments and wrote part of and corrected the manuscript.

### Conflict of interest statement

The authors declare that the research was conducted in the absence of any commercial or financial relationships that could be construed as a potential conflict of interest.
