# Supplementary material for: Maximization of Markers Linked in Coupling for Tetraploid Potatoes via Monoparental Haploids
Source: Front Plant Sci. 2018 May 7;9:620. doi: 10.3389/fpls.2018.00620 (PMC5949705; doi:10.3389/fpls.2018.00620)
Supplement: Supplementary file 1 [file Data_Sheet_1.DOCX]

***Supplementary Material***

**Maximization of Markers Linked in Coupling for Tetraploid Potatoes via Monoparental Dihaploids**

**Annette Bartkiewicz^1^, Friederike Chilla^1^, Diro Terefe-Ayana^2^, Jens Lübeck^3^, Josef Strahwald^3^, Eckhard Tacke^4^, Hans-Reinhard Hofferbert^4^, Marcus Linde^1^, Thomas Debener^1^***

^1^ Institute of Plant Genetics, Department of Molecular Plant Breeding, Leibniz University Hannover, Hannover, Germany

^2^ Westhoff, Südlohn-Oeding, Germany

^3^ SaKa Pflanzenzucht GmbH & Co. KG, Windeby, Germany

^4^ Böhm-Nordkartoffel Agrarproduktion GmbH & Co. OHG, Ebstorf, Germany

*** Correspondence:**

Prof. Dr. Thomas Debener

**Table S1: SNP markers revealing a putative *S. phureja* introgression in the dihaploid progeny for the crosses P208 x *S. phureja* IVP101 (A), P208 x *S. phureja* IVP35 (B) and P809 x *S. phureja* IVP101 (C).** Altogether, twelve SNPs for each of the crosses with P208 and 27 SNPs for the cross with P809 were identified. The number and percentages of putatively introgressed genotypes is given for each SNP, as well as the chromosome on which the marker is located and the chromosome position in the potato genome.

| A | **SNP marker** | **genotype P208** | **genotype *S. phureja* IVP101** | **number of genotypes with *S. phureja* introgression** | **percentage of genotypes with *S. phureja* introgression** | **chromosome** | **position according to genome browser** |
| --- | --- | --- | --- | --- | --- | --- | --- |
|  | solcap_snp_c2_52712 | BB | AB | 61 | 54.95 % | 1 | 8,645,583 |
|  | solcap_snp_c2_25560 | AA | BB | 19 | 17.12 % | 3 | 45,904,981 |
|  | solcap_snp_c2_21604 | BB | AB | 1 | 0.90 % | 4 | 22,069,806 |
|  | solcap_snp_c2_35942 | BB | AA | 35 | 31.53 % | 4 | 70,878,916 |
|  | solcap_snp_c2_42407 | BB | AB | 52 | 46.85 % | 5 | 49,094,535 |
|  | solcap_snp_c2_3451 | BB | AB | 13 | 11.71 % | 5 | 51,697,156 |
|  | solcap_snp_c2_12400 | BB | AB | 8 | 7.21 % | 6 | 24,508,161 |
|  | solcap_snp_c2_49839 | BB | AA | 12 | 10.81 % | 7 | 10,163,376 |
|  | solcap_snp_c2_49603 | AA | BB | 2 | 1.80 % | 8 | 49,813,597 |
|  | solcap_snp_c2_10957 | BB | AA | 64 | 57.66 % | 9 | 2,083,602 |
|  | solcap_snp_c2_41768 | BB | AA | 51 | 45.95 % | 10 | 59,125,011 |
|  | solcap_snp_c2_54921 | AA | BB | 17 | 15.32 % | - | - |
| B | **SNP marker** | **genotype P208** | **genotype *S. phureja* IVP35** | **number of genotypes with *S. phureja* introgression** | **percentage of genotypes with *S. phureja* introgression** | **chromosome** | **position according to genome browser** |
|  | solcap_snp_c2_52712 | BB | AB | 70 | 66.04 % | 1 | 8,645,583 |
|  | solcap_snp_c1_7325 | AA | BB | 1 | 0.94 % | 2 | 42,764,457 |
|  | solcap_snp_c2_25560 | AA | BB | 21 | 19.81 % | 3 | 45,904,981 |
|  | solcap_snp_c2_35942 | BB | AB | 41 | 38.68 % | 4 | 70,878,916 |
|  | solcap_snp_c2_42407 | BB | AB | 52 | 49.06 % | 5 | 49,094,535 |
|  | solcap_snp_c2_3451 | BB | AB | 7 | 6.60 % | 5 | 51,697,156 |
|  | solcap_snp_c2_49839 | BB | AA | 13 | 12.26 % | 7 | 10,163,376 |
|  | solcap_snp_c2_10957 | BB | AA | 51 | 48.11 % | 9 | 2,083,602 |
|  | solcap_snp_c2_41768 | BB | AA | 48 | 45.28 % | 10 | 59,125,011 |
|  | solcap_snp_c2_32982 | AA | BB | 26 | 24.53 % | 11 | 9,734,854 |
|  | solcap_snp_c2_17747 | BB | AB | 16 | 15.09 % | 12 | 50,572,755 |
|  | solcap_snp_c2_54921 | AA | BB | 17 | 16.04 % | - | - |
| C | **SNP marker** | **genotype P809** | **genotype *S. phureja* IVP101** | **number of genotypes with *S. phureja* introgression** | **percentage of genotypes with *S. phureja* introgression** | **chromosome** | **position according to genome browser** |
|  | solcap_snp_c1_12296 | AA | AB | 5 | 12.82 % | 2 | 15,287,033 |
|  | solcap_snp_c2_27271 | AA | BB | 23 | 58.97 % | 2 | 44,458,744 |
|  | solcap_snp_c2_25560 | AA | BB | 9 | 23.08 % | 3 | 45,904,981 |
|  | solcap_snp_c1_7569 | BB | AA | 23 | 58.97 % | 4 | 660,102 |
|  | solcap_snp_c2_26773 | BB | AA | 18 | 46.15 % | 4 | 9,276,426 |
|  | solcap_snp_c2_21604 | BB | AB | 5 | 12.82 % | 4 | 22,069,806 |
|  | solcap_snp_c1_3311 | AA | AB | 19 | 48.72 % | 4 | 46,475,704 |
|  | solcap_snp_c2_36053 | AA | BB | 20 | 51.28 % | 4 | 58,951,832 |
|  | solcap_snp_c2_23728 | BB | AA | 19 | 48.72 % | 5 | 325,019 |
|  | solcap_snp_c2_53223 | BB | AA | 14 | 35.90 % | 5 | 20,390,111 |
|  | solcap_snp_c2_5219 | BB | AB | 20 | 51.28 % | 5 | 42,524,322 |
|  | solcap_snp_c1_13636 | AA | AB | 27 | 69.23 % | 6 | 1,877,188 |
|  | solcap_snp_c2_27606 | AA | BB | 16 | 41.03 % | 6 | 3,440,844 |
|  | solcap_snp_c2_49839 | BB | AA | 12 | 30.77 % | 7 | 10,163,376 |
|  | solcap_snp_c2_51954 | BB | AB | 14 | 35.90 % | 8 | 1,868,572 |
|  | solcap_snp_c2_29491 | BB | AA | 24 | 61.54 % | 8 | 26,141,458 |
|  | solcap_snp_c1_6140 | AA | BB | 22 | 56.41 % | 8 | 29,813,975 |
|  | solcap_snp_c2_44299 | BB | AB | 12 | 30.77 % | 8 | 45,631,066 |
|  | solcap_snp_c1_5559 | BB | AB | 20 | 51.28 % | 8 | 55,114,819 |
|  | solcap_snp_c1_14388 | BB | AA | 16 | 41.03 % | 9 | 650,478 |
|  | solcap_snp_c2_3010 | BB | AB | 15 | 38.46 % | 9 | 58,670,590 |
|  | solcap_snp_c2_55972 | BB | AA | 15 | 38.46 % | 11 | 3,182,994 |
|  | solcap_snp_c2_49299 | AA | BB | 3 | 7.69 % | 11 | 9,191,145 |
|  | solcap_snp_c2_17617 | AA | BB | 21 | 53.85 % | 12 | 50,382,217 |
|  | solcap_snp_c2_54921 | AA | BB | 1 | 2.56 % | - | - |
|  | solcap_snp_c2_52621 | BB | AB | 7 | 17.95 % | - | - |
|  | solcap_snp_c2_54069 | BB | AA | 17 | 43.59 % | - | - |

**Table S2: Overlapping genotypes with an *S. phureja* introgression.** Markers and their position are listed for the respective crosses as well as the number of introgressed genotypes and the number of genotypes that show an introgression for both markers of the respective marker combination.

| **Cross** | **Chromosome** | **Marker** | **Position** | **Introgressed Genotypes** | **Marker** | **Position** | **Introgressed Genotypes** | **Overlapping genotypes with introgression** |
| --- | --- | --- | --- | --- | --- | --- | --- | --- |
| P208 x *S. phureja* IVP101 | 4 | solcap_snp_c2_21604 | 22,069,806 | 1 | solcap_snp_c2_35942 | 70,878,916 | 35 | **0** |
| P208 x *S. phureja* IVP101 | 5 | solcap_snp_c2_42407 | 49,094,535 | 52 | solcap_snp_c2_3451 | 51,697,156 | 13 | **9** |
| P208 x *S. phureja* IVP35 | 5 | solcap_snp_c2_42407 | 49,094,535 | 52 | solcap_snp_c2_3451 | 51,697,156 | 7 | **5** |
| P809 x *S. phureja* IVP101 | 2 | solcap_snp_c1_12296 | 15,287,033 | 5 | solcap_snp_c2_27271 | 44,458,744 | 23 | **3** |
| P809 x *S. phureja* IVP101 | 4 | solcap_snp_c1_7569 | 660,102 | 23 | solcap_snp_c2_26773 | 9,276,426 | 18 | **9** |
| P809 x *S. phureja* IVP101 | 4 | solcap_snp_c1_7569 | 660,102 | 23 | solcap_snp_c2_21604 | 22,069,806 | 5 | **3** |
| P809 x *S. phureja* IVP101 | 4 | solcap_snp_c1_7569 | 660,102 | 23 | solcap_snp_c1_3311 | 46,475,704 | 19 | **9** |
| P809 x *S. phureja* IVP101 | 4 | solcap_snp_c1_7569 | 660,102 | 23 | solcap_snp_c2_36053 | 58,951,832 | 20 | **11** |
| P809 x *S. phureja* IVP101 | 4 | solcap_snp_c2_26773 | 9,276,426 | 18 | solcap_snp_c2_21604 | 22,069,806 | 5 | **5** |
| P809 x *S. phureja* IVP101 | 4 | solcap_snp_c2_26773 | 9,276,426 | 18 | solcap_snp_c1_3311 | 46,475,704 | 19 | **18** |
| P809 x *S. phureja* IVP101 | 4 | solcap_snp_c2_26773 | 9,276,426 | 18 | solcap_snp_c2_36053 | 58,951,832 | 20 | **9** |
| P809 x *S. phureja* IVP101 | 4 | solcap_snp_c2_21604 | 22,069,806 | 5 | solcap_snp_c1_3311 | 46,475,704 | 19 | **5** |
| P809 x *S. phureja* IVP101 | 4 | solcap_snp_c2_21604 | 22,069,806 | 5 | solcap_snp_c2_36053 | 58,951,832 | 20 | **2** |
| P809 x *S. phureja* IVP101 | 4 | solcap_snp_c1_3311 | 46,475,704 | 19 | solcap_snp_c2_36053 | 58,951,832 | 20 | **9** |
| P809 x *S. phureja* IVP101 | 5 | solcap_snp_c2_23728 | 325,019 | 19 | solcap_snp_c2_53223 | 20,390,111 | 14 | **9** |
| P809 x *S. phureja* IVP101 | 5 | solcap_snp_c2_23728 | 325,019 | 19 | solcap_snp_c2_5219 | 42,524,322 | 20 | **10** |
| P809 x *S. phureja* IVP101 | 5 | solcap_snp_c2_53223 | 20,390,111 | 14 | solcap_snp_c2_5219 | 42,524,322 | 20 | **5** |
| P809 x *S. phureja* IVP101 | 6 | solcap_snp_c1_13636 | 1,877,188 | 27 | solcap_snp_c2_27606 | 3,440,844 | 16 | **10** |
| P809 x *S. phureja* IVP101 | 8 | solcap_snp_c2_51954 | 1,868,572 | 14 | solcap_snp_c2_29491 | 26,141,458 | 24 | **5** |
| P809 x *S. phureja* IVP101 | 8 | solcap_snp_c2_51954 | 1,868,572 | 14 | solcap_snp_c1_6140 | 29,813,975 | 22 | **4** |
| P809 x *S. phureja* IVP101 | 8 | solcap_snp_c2_51954 | 1,868,572 | 14 | solcap_snp_c2_44299 | 45,631,066 | 12 | **4** |
| P809 x *S. phureja* IVP101 | 8 | solcap_snp_c2_51954 | 1,868,572 | 14 | solcap_snp_c1_5559 | 55,114,819 | 20 | **5** |
| P809 x *S. phureja* IVP101 | 8 | solcap_snp_c2_29491 | 26,141,458 | 24 | solcap_snp_c1_6140 | 29,813,975 | 22 | **22** |
| P809 x *S. phureja* IVP101 | 8 | solcap_snp_c2_29491 | 26,141,458 | 24 | solcap_snp_c2_44299 | 45,631,066 | 12 | **9** |
| P809 x *S. phureja* IVP101 | 8 | solcap_snp_c2_29491 | 26,141,458 | 24 | solcap_snp_c1_5559 | 55,114,819 | 20 | **13** |
| P809 x *S. phureja* IVP101 | 8 | solcap_snp_c1_6140 | 29,813,975 | 22 | solcap_snp_c2_44299 | 45,631,066 | 12 | **9** |
| P809 x *S. phureja* IVP101 | 8 | solcap_snp_c1_6140 | 29,813,975 | 22 | solcap_snp_c1_5559 | 55,114,819 | 20 | **12** |
| P809 x *S. phureja* IVP101 | 8 | solcap_snp_c2_44299 | 45,631,066 | 12 | solcap_snp_c1_5559 | 55,114,819 | 20 | **7** |
| P809 x *S. phureja* IVP101 | 9 | solcap_snp_c1_14388 | 650,478 | 16 | solcap_snp_c2_3010 | 58,670,590 | 15 | **8** |
| P809 x *S. phureja* IVP101 | 11 | solcap_snp_c2_55972 | 3,182,994 | 15 | solcap_snp_c2_49299 | 9,191,145 | 3 | **2** |

**Table S3: Putative introgression of *S. phureja* genome in the dihaploid progeny.** The number of SNPs showing an allele configuration that is specific for the pollinator as well as the number of genotypes showing this allele configuration and the percentage of the pollinator genome within the progeny are listed for the crosses P208 x *S. phureja* IVP101 (A), P208 x *S.phureja* IVP35 (B) and P809 x *S. phureja* IVP101 (C).

| **A** | **Number of *S. phureja* IVP101-specific SNPs in dihaploid progeny of the P208 population** | **Number of genotypes in the P208 population showing *S. phureja* IVP101-specific allele configuration** | **Percentage of *S. phureja* genome in the individual genotypes [%]** |
| --- | --- | --- | --- |
|  | 0 | 1 | 0.0 |
|  | 1 | 10 | 0.155 |
|  | 2 | 23 | 0.309 |
|  | 3 | 26 | 0.464 |
|  | 4 | 27 | 0.618 |
|  | 5 | 14 | 0.773 |
|  | 6 | 8 | 0.927 |
|  | 7 | 1 | 1.082 |
|  | 8 | 1 | 1.236 |
| **B** | **Number of *S. phureja* IVP35-specific SNPs in dihaploid progeny of the P208 population** | **Number of genotypes in the P208 population showing *S. phureja* IVP35-specific allele configuration** | **Percentage of *S. phureja* genome in the individual genotypes [%]** |
|  | 0 | 1 | 0.0 |
|  | 1 | 12 | 0.158 |
|  | 2 | 14 | 0.316 |
|  | 3 | 22 | 0.474 |
|  | 4 | 29 | 0.632 |
|  | 5 | 19 | 0.790 |
|  | 6 | 4 | 0.948 |
|  | 7 | 2 | 1.106 |
|  | 8 | 1 | 1.264 |
| **C** | **Number of *S. phureja* IVP101-specific SNPs in dihaploid progeny of the P809 population** | **Number of genotypes in the P809 population showing *S. phureja* IVP101-specific allele configuration** | **Percentage of *S. phureja* genome in the individual genotypes [%]** |
|  | 5 | 1 | 0.629 |
|  | 7 | 4 | 0.881 |
|  | 8 | 5 | 1.006 |
|  | 9 | 2 | 1.132 |
|  | 10 | 6 | 1.258 |
|  | 11 | 6 | 1.384 |
|  | 12 | 2 | 1.509 |
|  | 13 | 8 | 1.635 |
|  | 14 | 3 | 1.761 |
|  | 15 | 2 | 1.887 |

**Table S4: Useful single dose markers in a cross between P208 and a second tetraploid parent.** The numbers of putative single dose markers in a biparental tetraploid cross are listed for 125 cultivars, as well as the percentages of useful single dose markers in a biparental cross when compared to the 2,548 single dose markers in the P208 population.

| **Tetraploid potato cultivar** | **number of SNPs that would segregate 1:1 in biparental population with P208** | **percentage of useful SNPs compared to the P208 population [%]** |
| --- | --- | --- |
| Adam | 839 | 32.93 |
| Agila | 800 | 31.40 |
| Agria | 712 | 27.94 |
| Alegria | 777 | 30.49 |
| Altesse | 793 | 31.12 |
| Altus | 921 | 36.15 |
| Amanda | 706 | 27.71 |
| Ametyst | 750 | 29.43 |
| Andante | 724 | 28.41 |
| Annalena | 738 | 28.96 |
| Antonia | 738 | 28.96 |
| Aruba | 802 | 31.48 |
| Avano | 805 | 31.59 |
| Avarna | 858 | 33.67 |
| Axion | 928 | 36.42 |
| Baltic Cream | 683 | 26.81 |
| Bartek | 697 | 27.35 |
| Bavapom | 699 | 27.43 |
| Belana | 718 | 28.18 |
| Bila | 773 | 30.34 |
| Birte | 866 | 33.99 |
| Borwina | 696 | 27.32 |
| Boryna | 779 | 30.57 |
| Bosman | 629 | 24.69 |
| Bryza | 707 | 27.75 |
| Burana | 842 | 33.05 |
| Campina | 775 | 30.42 |
| Caruso | 762 | 29.91 |
| Cedron | 634 | 24.88 |
| Cekin | 783 | 30.73 |
| Combi | 657 | 25.78 |
| Concordia | 773 | 30.34 |
| Cumbica | 686 | 26.92 |
| Deodara | 543 | 21.31 |
| Desirée | 609 | 23.90 |
| Django | 777 | 30.49 |
| Etiuda | 635 | 24.92 |
| Eurobravo | 745 | 29.24 |
| Finezja | 708 | 27.79 |
| Finka | 739 | 29.00 |
| Francisca | 751 | 29.47 |
| Gandawa | 632 | 24.80 |
| Gawin | 714 | 28.02 |
| Glada | 747 | 29.32 |
| Goldmarie | 717 | 28.14 |
| Gustaw | 822 | 32.26 |
| Gwiazda | 755 | 29.63 |
| Harpun | 696 | 27.32 |
| Heidi | 769 | 30.18 |
| Hubal | 722 | 28.34 |
| Ibis | 781 | 30.65 |
| Ignacy | 703 | 27.59 |
| Igor | 829 | 32.54 |
| Ikar | 790 | 31.00 |
| Inwestor | 694 | 27.24 |
| Irga | 711 | 27.90 |
| Ivetta | 781 | 30.65 |
| Jasia | 688 | 27.00 |
| Jubilat | 682 | 26.77 |
| Jurek | 761 | 29.87 |
| Justa | 725 | 28.45 |
| Jutrzenka | 751 | 29.47 |
| Juwel | 810 | 31.79 |
| Kaszub | 840 | 32.97 |
| Krone | 763 | 29.95 |
| Kuba | 820 | 32.18 |
| Kuras | 699 | 27.43 |
| Laura | 782 | 30.69 |
| Legenda | 772 | 30.30 |
| Lilly | 720 | 28.26 |
| Logo | 861 | 33.79 |
| Lord | 770 | 30.22 |
| Lyoness | 844 | 33.12 |
| Marabel | 801 | 31.44 |
| Marena | 783 | 30.73 |
| Mariola | 868 | 34.07 |
| Megusta | 748 | 29.36 |
| Merano | 799 | 31.36 |
| Michalina | 746 | 29.28 |
| Milek | 814 | 31.95 |
| Miriam | 777 | 30.49 |
| Montana | 806 | 31.63 |
| Natascha | 767 | 30.10 |
| Oberon | 822 | 32.26 |
| Oman | 863 | 33.87 |
| Opal | 728 | 28.57 |
| Osira | 806 | 31.63 |
| Ottawa | 630 | 24.73 |
| Owacja | 631 | 24.76 |
| Panda | 815 | 31.99 |
| Pasat | 729 | 28.61 |
| Pasja | 808 | 31.71 |
| Pokusa | 761 | 29.87 |
| Promyk | 809 | 31.75 |
| Quadriga | 863 | 33.87 |
| Renate | 845 | 33.16 |
| Romanza | 770 | 30.22 |
| Roncalla | 747 | 29.32 |
| Rudawa | 718 | 28.18 |
| Salute | 829 | 32.54 |
| Saphir | 798 | 31.32 |
| Sekwana | 675 | 26.49 |
| Seresta | 863 | 33.87 |
| Sevim | 705 | 27.67 |
| Skawa | 806 | 31.63 |
| Sleza | 715 | 28.06 |
| Sonda | 662 | 25.98 |
| Soraya | 794 | 31.16 |
| Stasia | 791 | 31.04 |
| Svenja | 703 | 27.59 |
| Syrena | 815 | 31.99 |
| Taifun | 853 | 33.48 |
| Talent | 713 | 27.98 |
| Tessa | 683 | 26.81 |
| Tetyda | 864 | 33.91 |
| Toccata | 691 | 27.12 |
| Tomensa | 786 | 30.85 |
| Toscana | 723 | 28.38 |
| Transit | 676 | 26.53 |
| Troja | 722 | 28.34 |
| Ulme | 713 | 27.98 |
| Valetta | 861 | 33.79 |
| Valenzia | 764 | 29.98 |
| Zagloba | 584 | 22.92 |
| Zenia | 776 | 30.46 |

**Table S5: Test for normal distribution of phenotypic data using a Shapiro-Wilk normality test.** Test statistics as well as p-values are listed for average shoot length, average number of nodes and average number of tubers before and after Box-Cox transformation of the phenotypic data. A p-value >0.05 indicates normal distribution of the phenotypic data.

| **Phenotypic trait** | **Shapiro-Wilk normality test with original data** | | **Shapiro-Wilk normality test with Box-Cox transformed data** | |
| --- | --- | --- | --- | --- |
|  | test statistic W | p-value | test statistic W | p-value |
| **Average shoot length** | 0.8831 | 3.306e-10 | 0.9807 | 0.01935 |
| **Average number of nodes** | 0.9882 | 0.1726 | 0.9929 | 0.5858 |
| **Average number of tubers** | 0.6354 | <2.2e-16 | 0.9909 | 0.3171 |
| **Average tuber weight** | 0.7313 | <2.2e-16 | 0.9914 | 0.3685 |

**Table S6: Test for correlation of phenotypic data using a Spearman-correlation test.** Correlation coefficients as well as p-values are listed for all phenotypic data after Box-Cox transformation.

| **Phenotypic traits** | **Correlation coefficient** | **p-value** |
| --- | --- | --- |
| **tuber weight – tuber number** | 0.6080252 | < 2.2e-16 |
| **tuber weight – shoot length** | 0.7502981 | < 2.2e-16 |
| **tuber weight – number of nodes** | 0.6723655 | < 2.2e-16 |
| **tuber number – shoot length** | 0.5933554 | < 2.2e-16 |
| **tuber number – number of nodes** | 0.57623 | 6.847e-16 |
| **shoot length – number of nodes** | 0.9131605 | < 2.2e-16 |


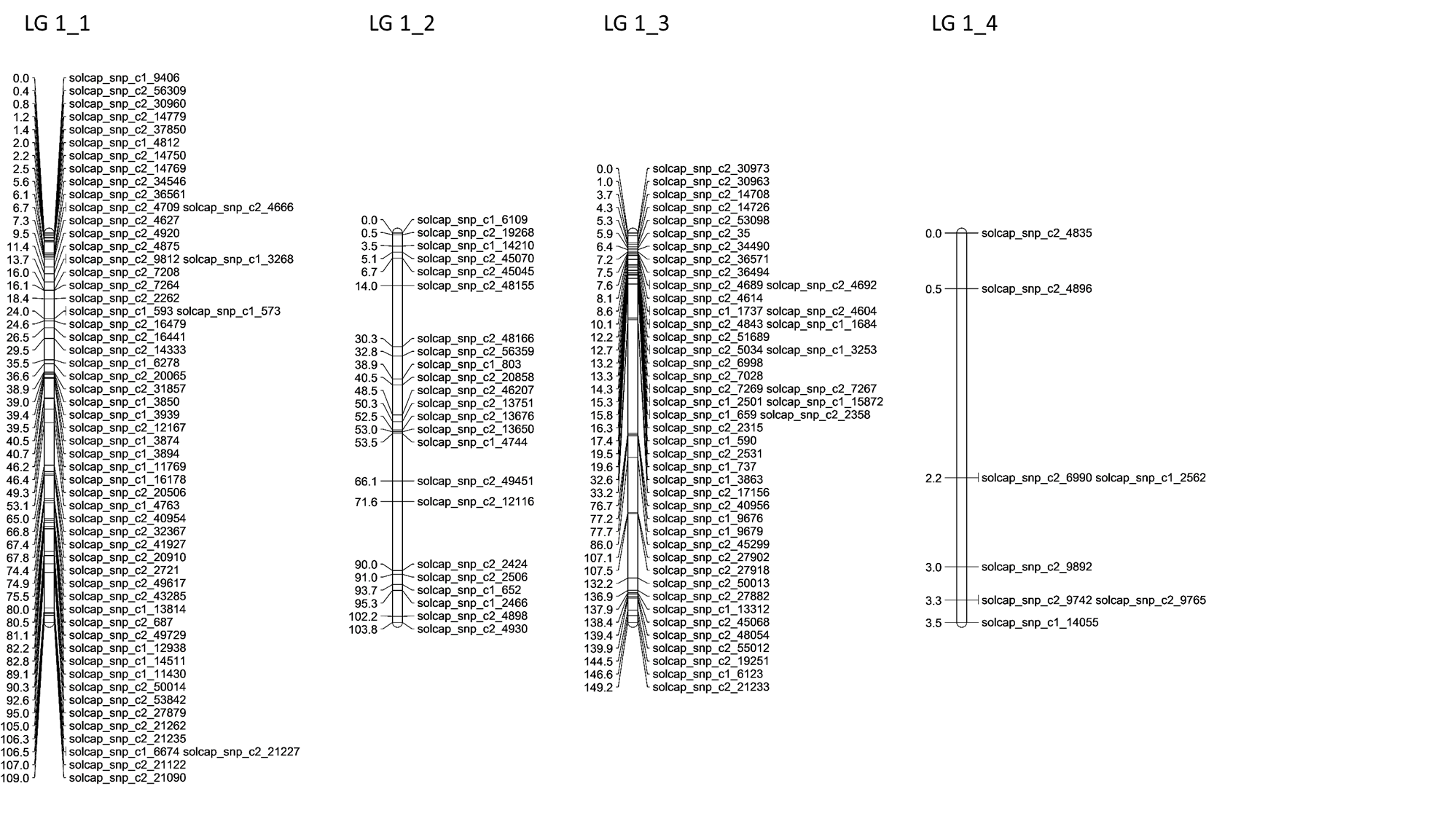


**Figure S1: Linkage groups constructed in JoinMap®4 with single dose SNP markers segregating in the P208 population.** The four linkage groups represent potato chromosome 1.


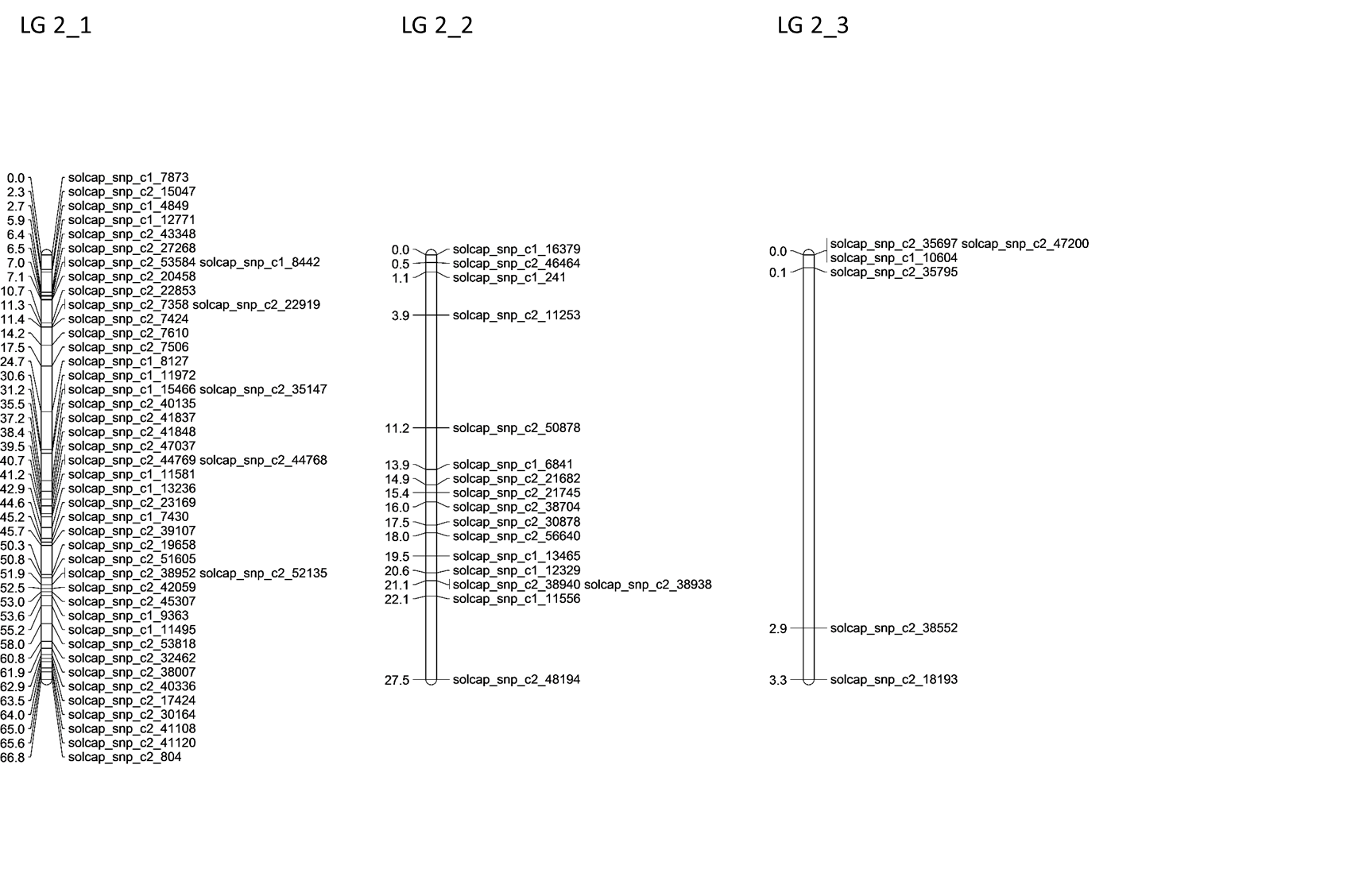


**Figure S2: Linkage groups constructed in JoinMap®4 with single dose SNP markers segregating in the P208 population.** The three linkage groups represent potato chromosome 2.


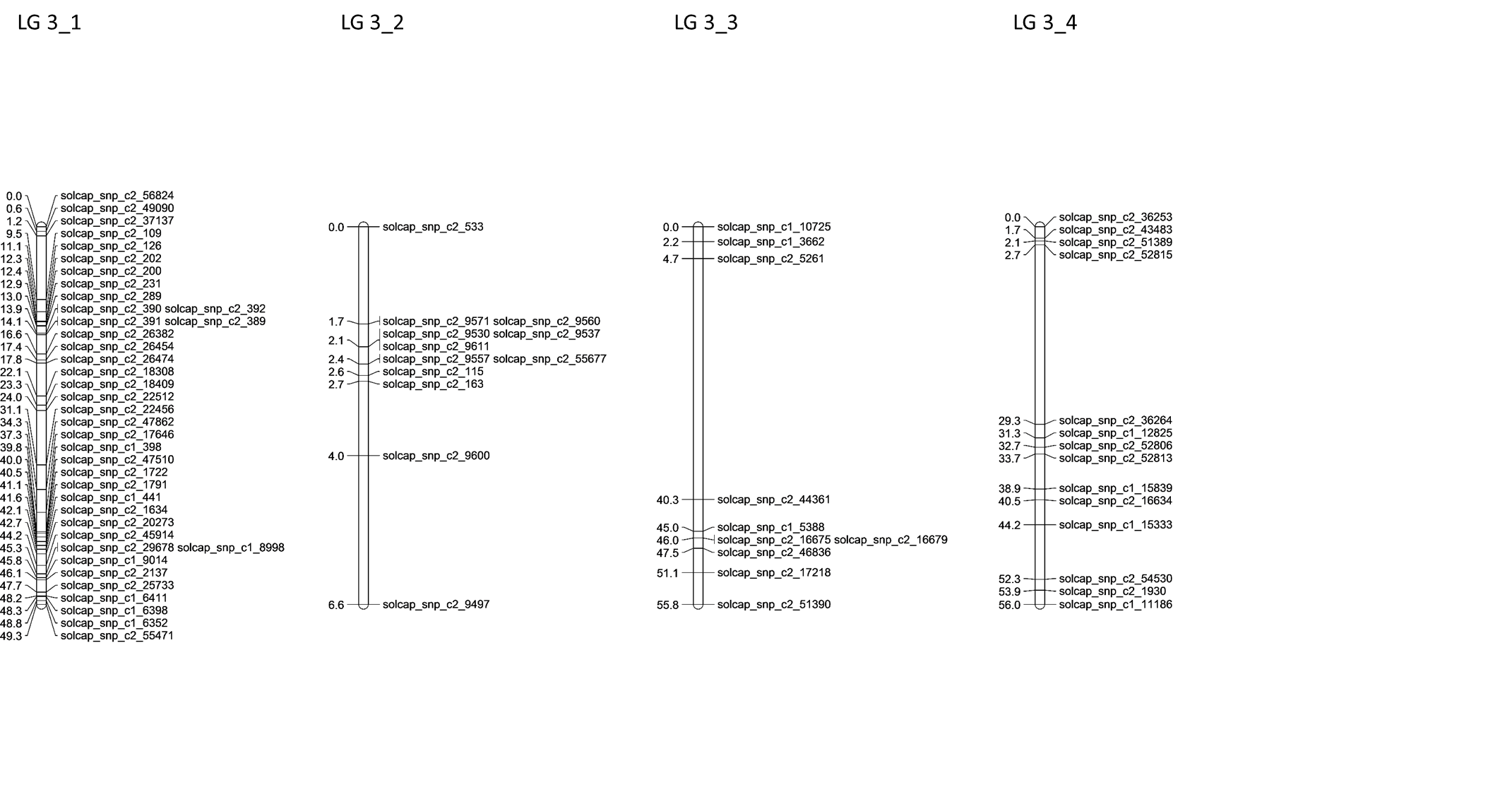
**Figure S3: Linkage groups constructed in JoinMap®4 with single dose SNP markers segregating in the P208 population.** The four linkage groups represent potato chromosome 3.


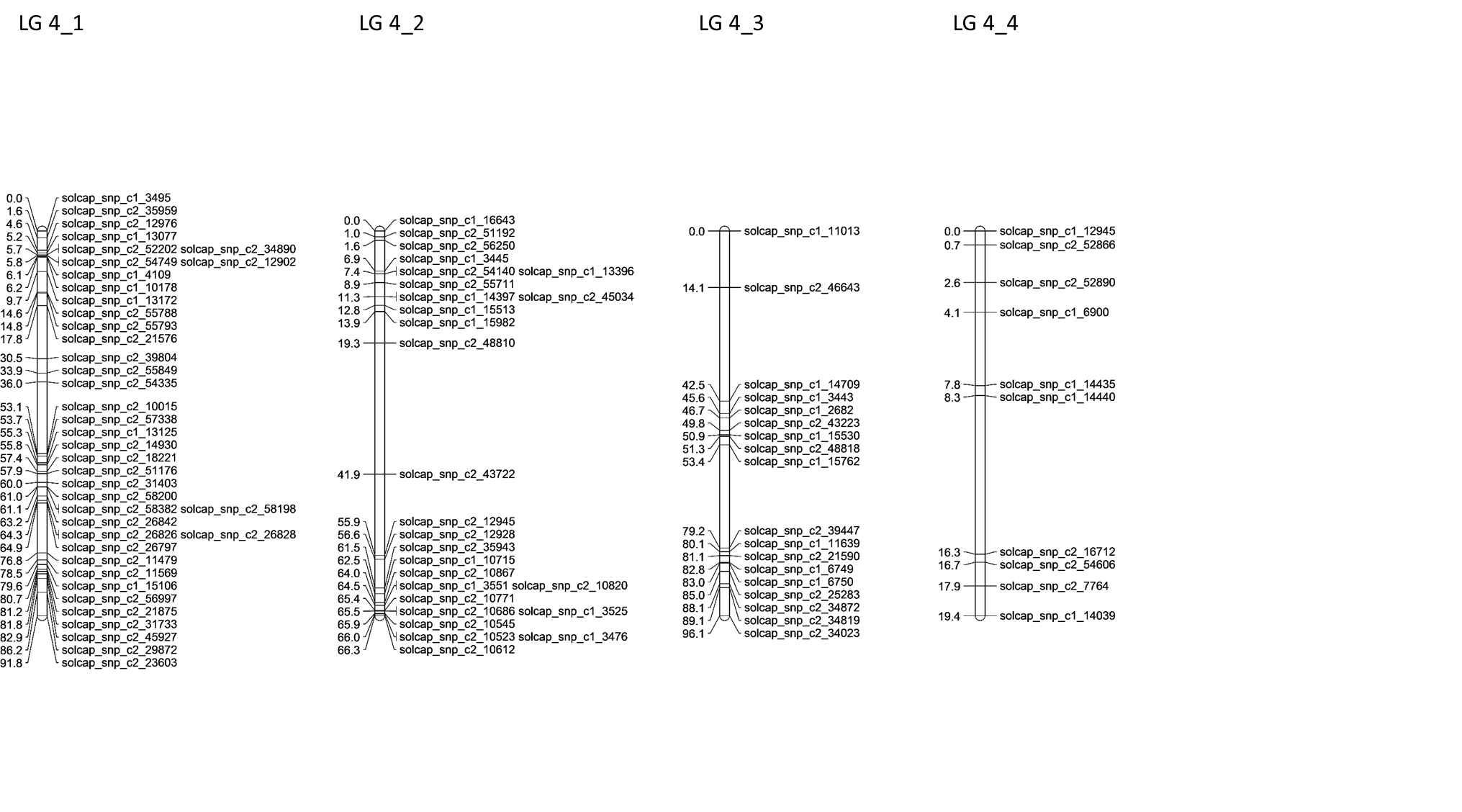


**Figure S4: Linkage groups constructed in JoinMap®4 with single dose SNP markers segregating in the P208 population.** The four linkage groups represent potato chromosome 4.


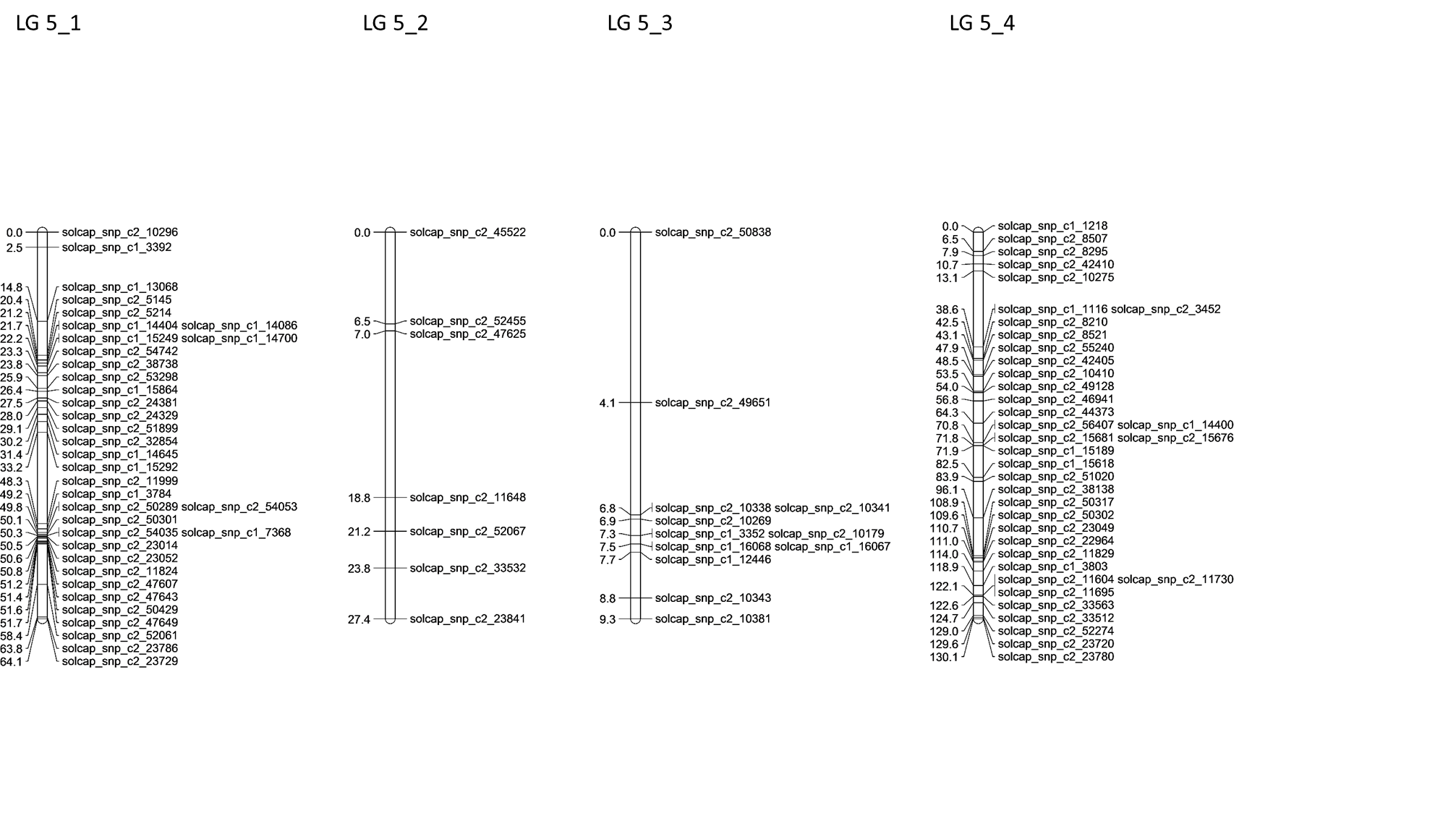


**Figure S5: Linkage groups constructed in JoinMap®4 with single dose SNP markers segregating in the P208 population.** The four linkage groups represent potato chromosome 5.


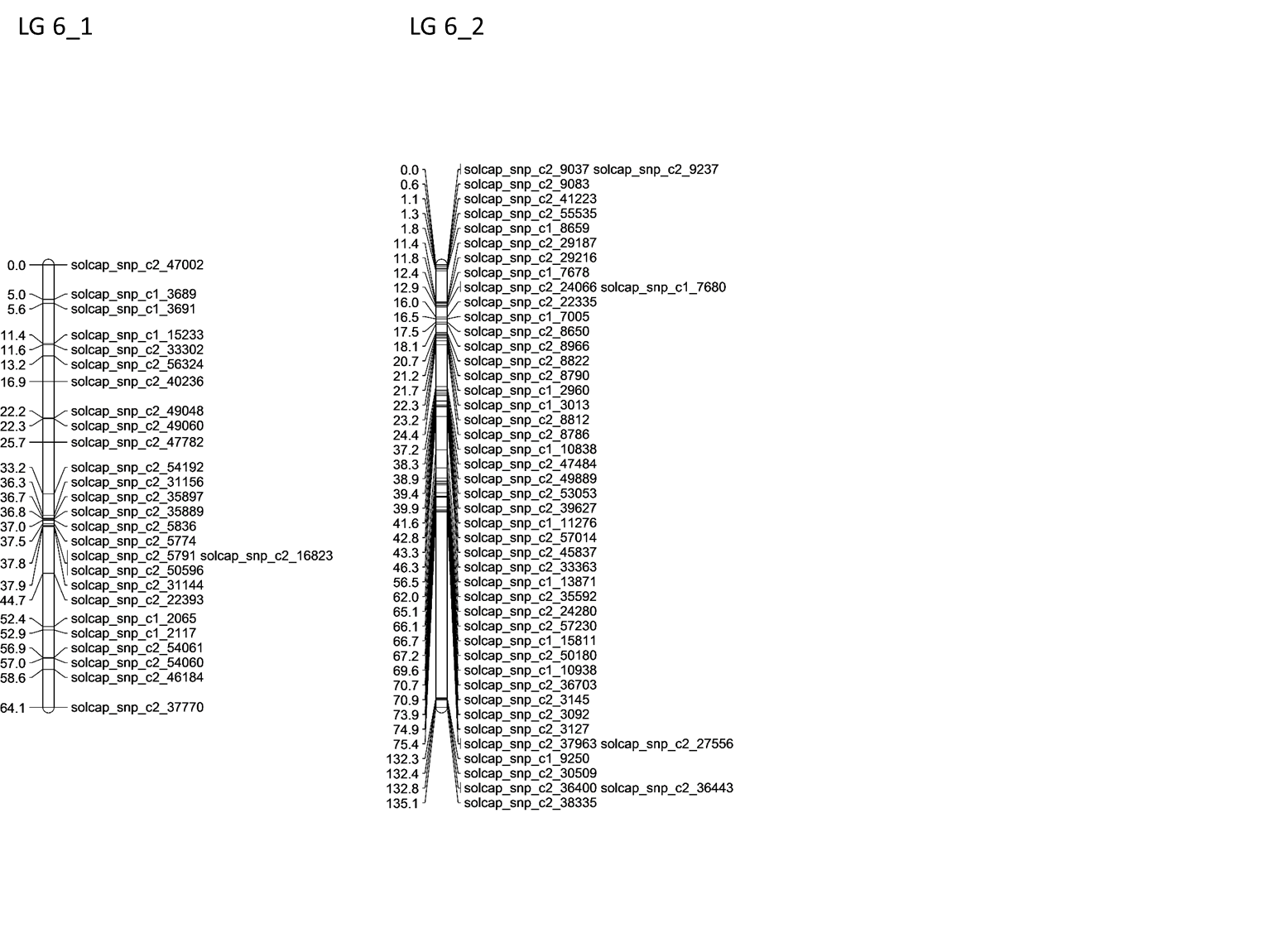


**Figure S6: Linkage groups constructed in JoinMap®4 with single dose SNP markers segregating in the P208 population.** The two linkage groups represent potato chromosome 6.


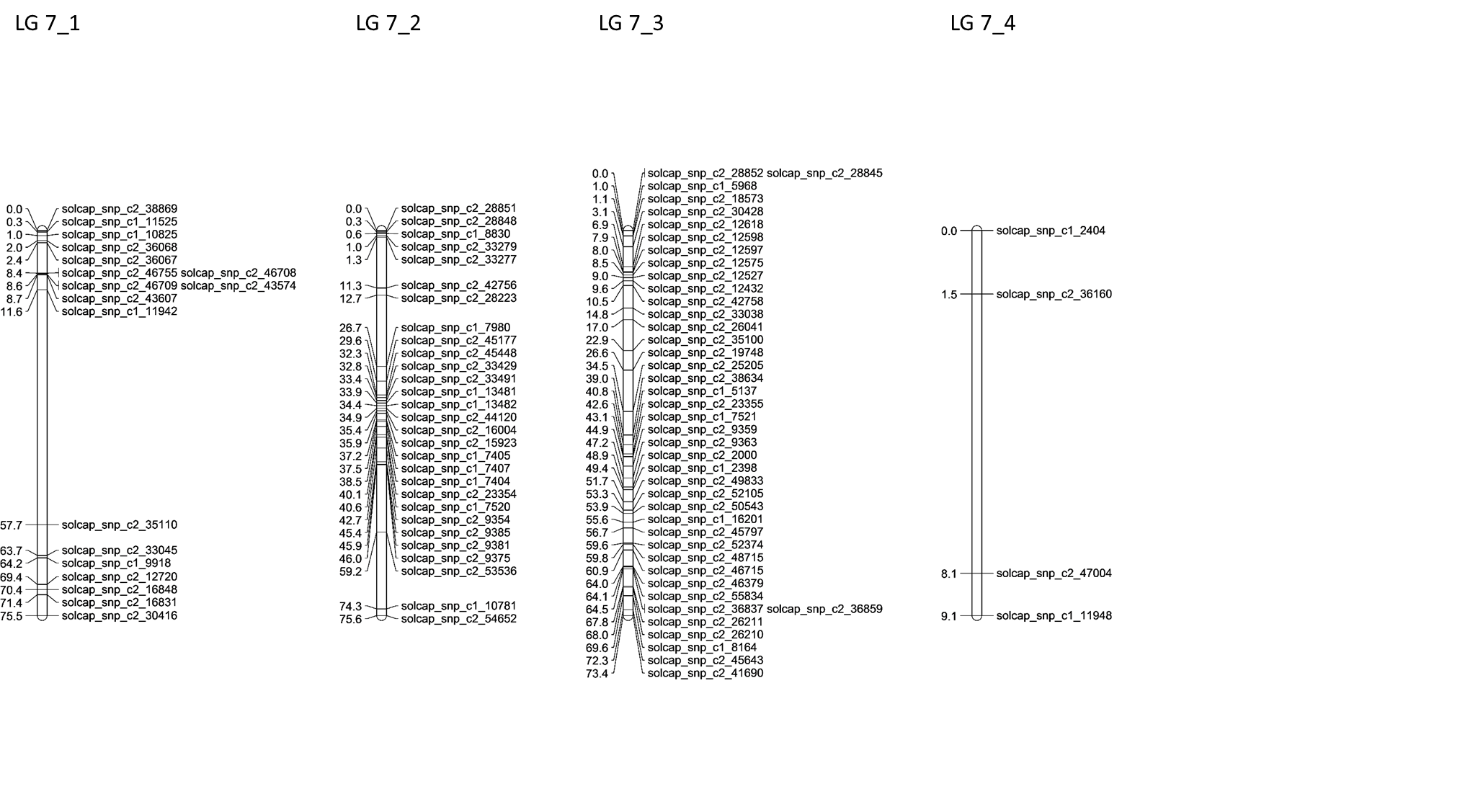


**Figure S7: Linkage groups constructed in JoinMap®4 with single dose SNP markers segregating in the P208 population.** The four linkage groups represent potato chromosome 7.


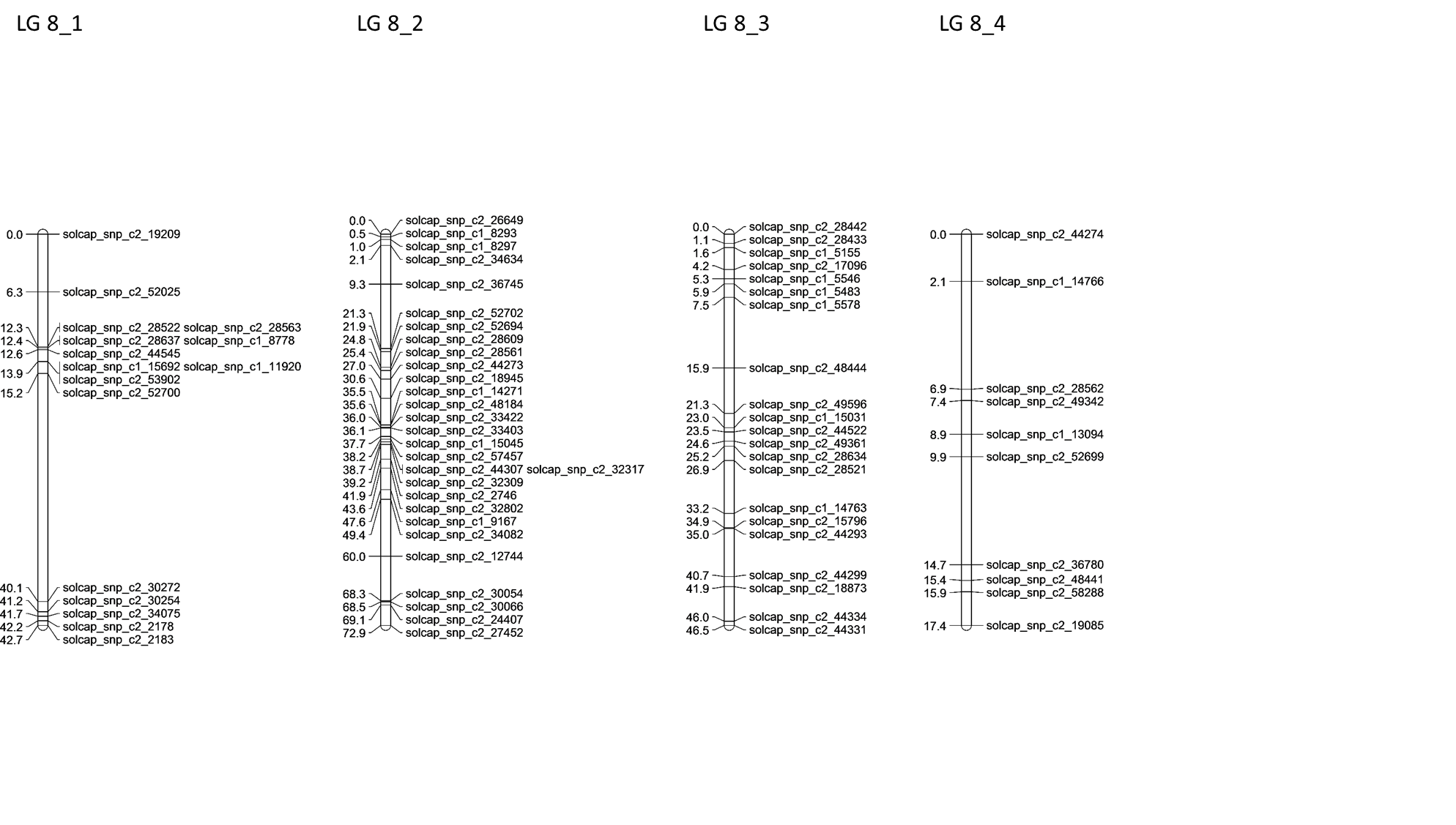


**Figure S8: Linkage groups constructed in JoinMap®4 with single dose SNP markers segregating in the P208 population.** The four linkage groups represent potato chromosome 8.


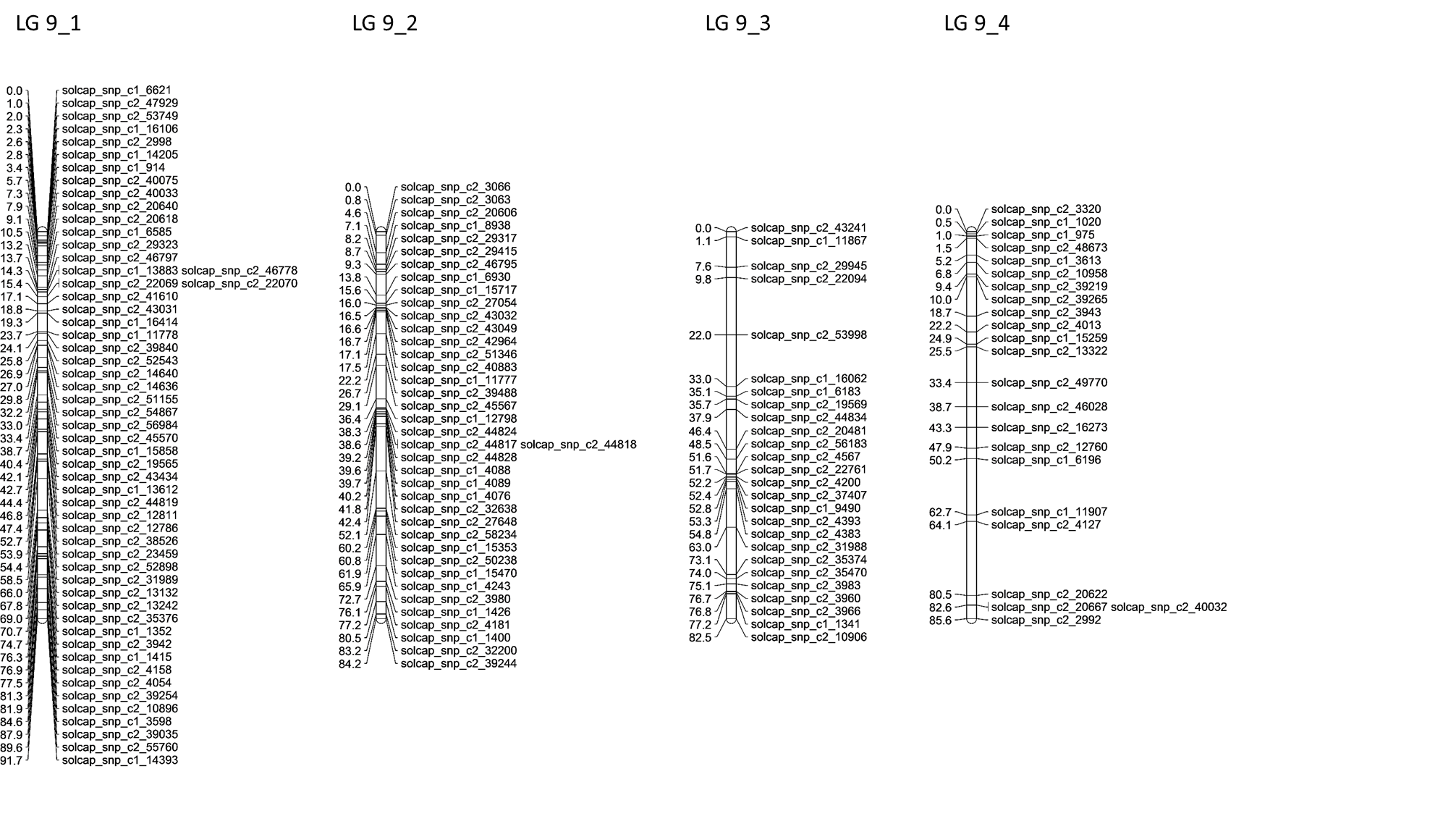


**Figure S9: Linkage groups constructed in JoinMap®4 with single dose SNP markers segregating in the P208 population.** The four linkage groups represent potato chromosome 9.


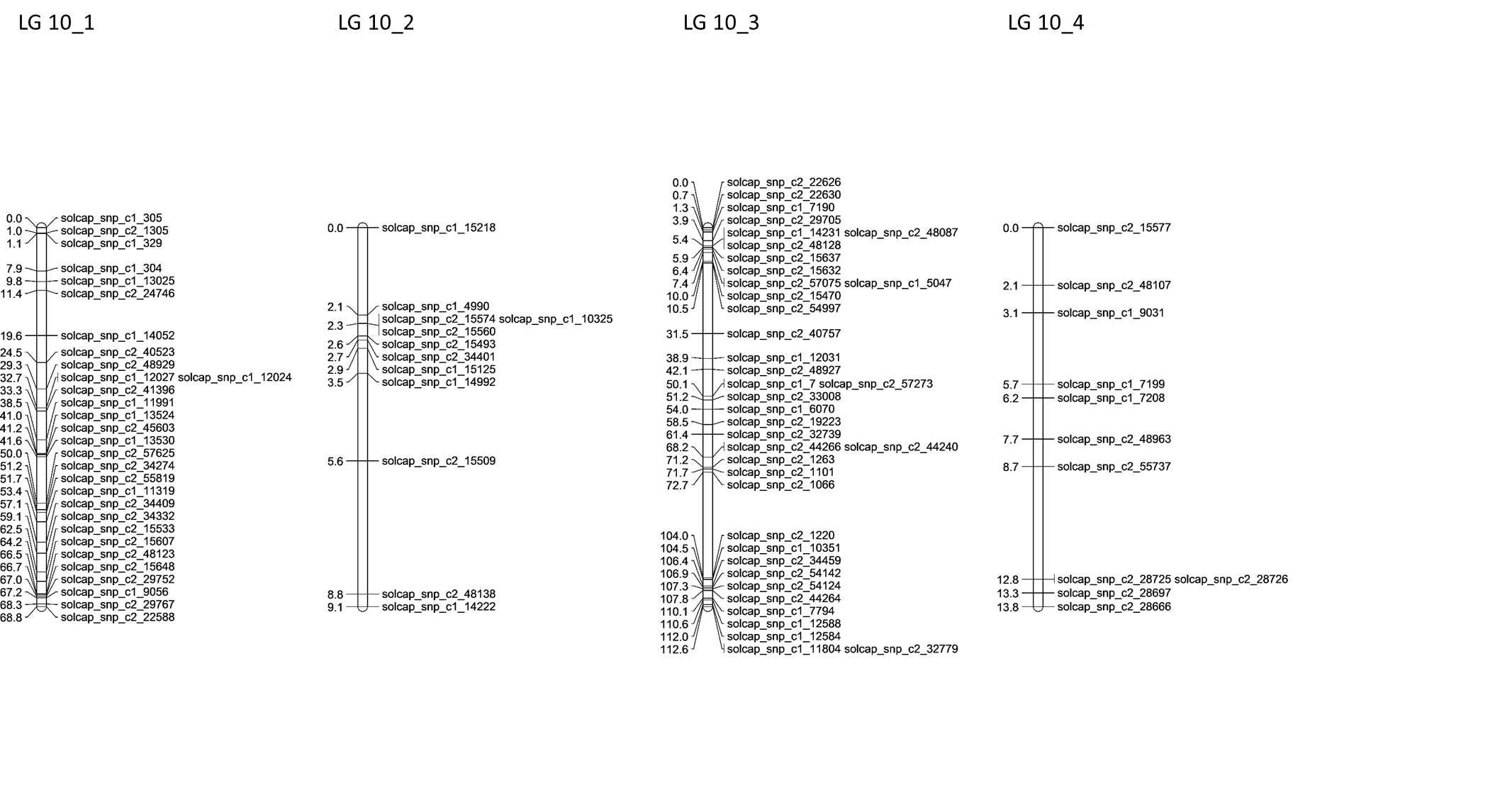


**Figure S10: Linkage groups constructed in JoinMap®4 with single dose SNP markers segregating in the P208 population.** The four linkage groups represent potato chromosome 10.


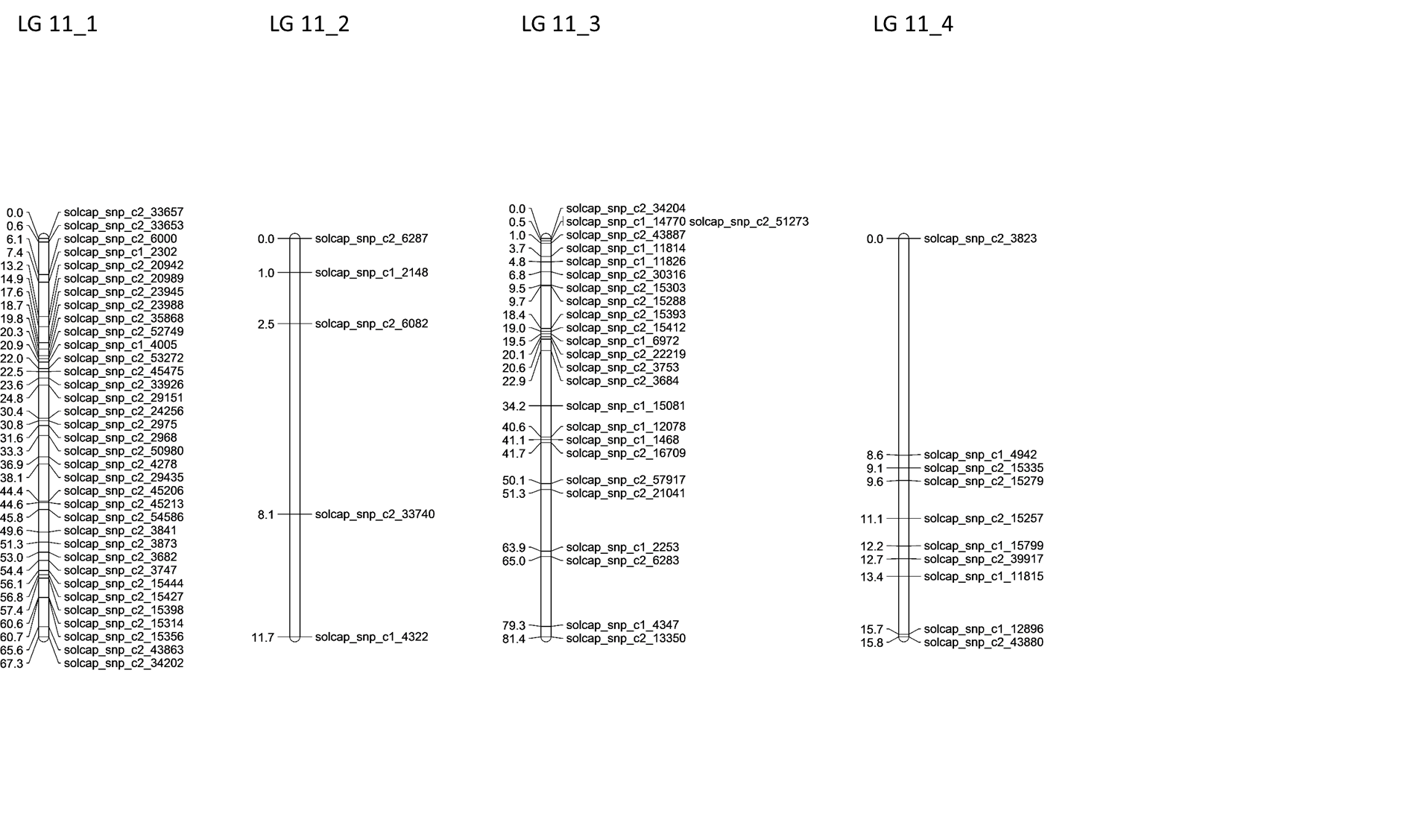


**Figure S11: Linkage groups constructed in JoinMap®4 with single dose SNP markers segregating in the P208 population.** The four linkage groups represent potato chromosome 11.


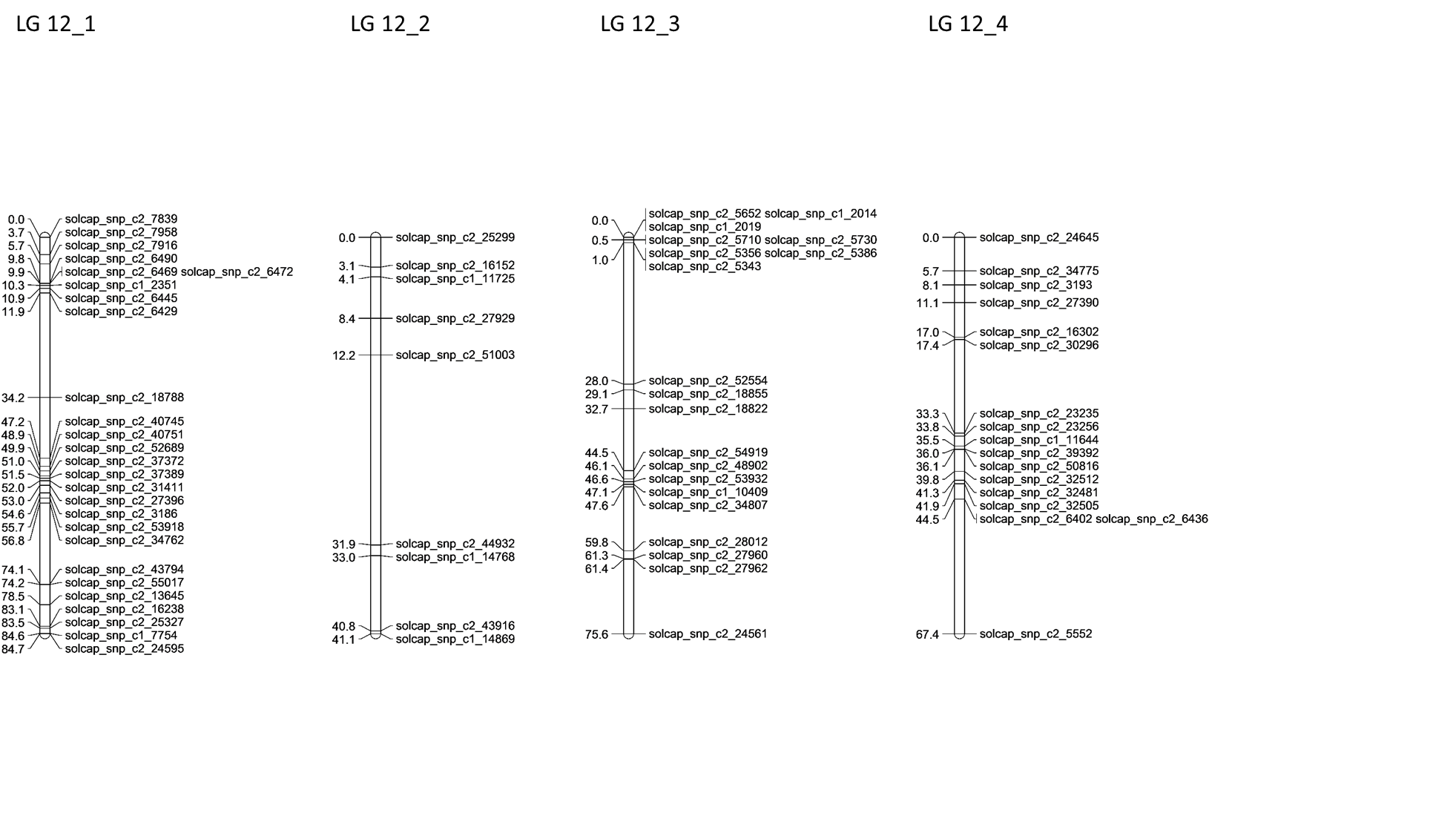


**Figure S12: Linkage groups constructed in JoinMap®4 with single dose SNP markers segregating in the P208 population.** The four linkage groups represent potato chromosome 12.


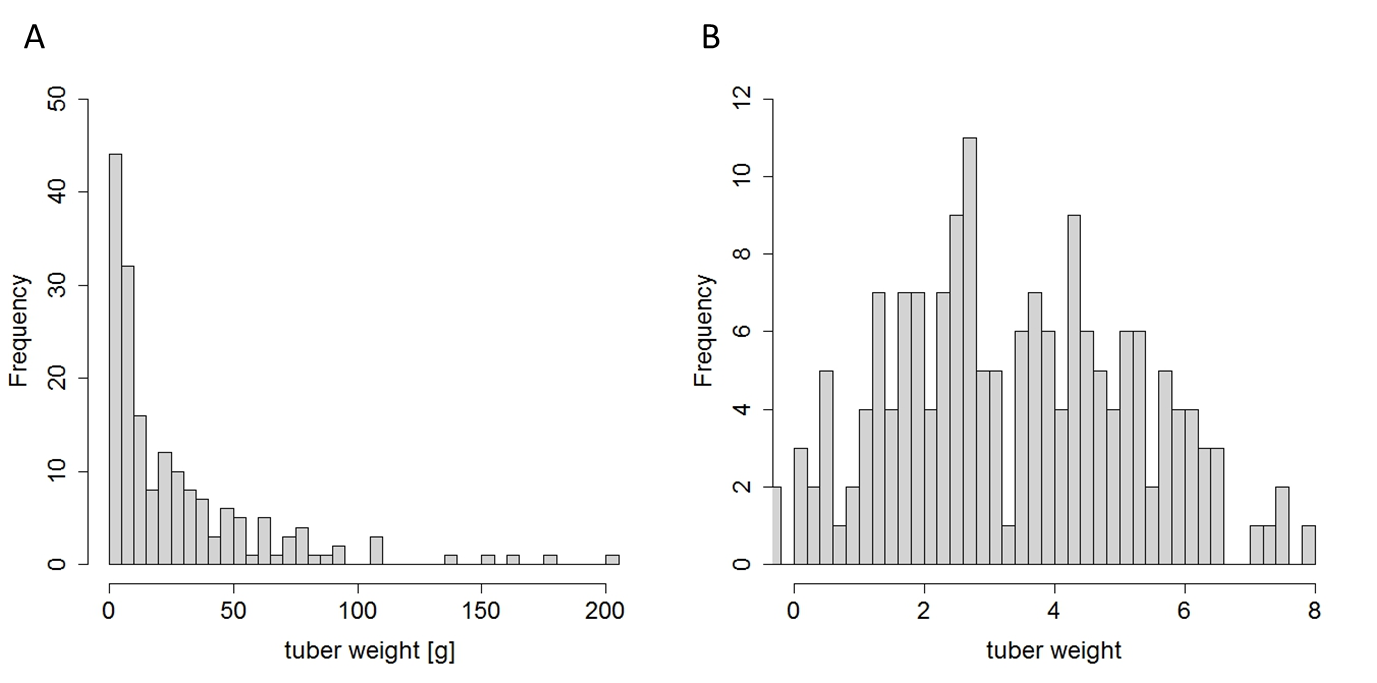


**Figure S13: Distribution of phenotypic data for average tuber weight.** Distributions are shown before (A) and after (B) Box-Cox transformation of the phenotypic data.


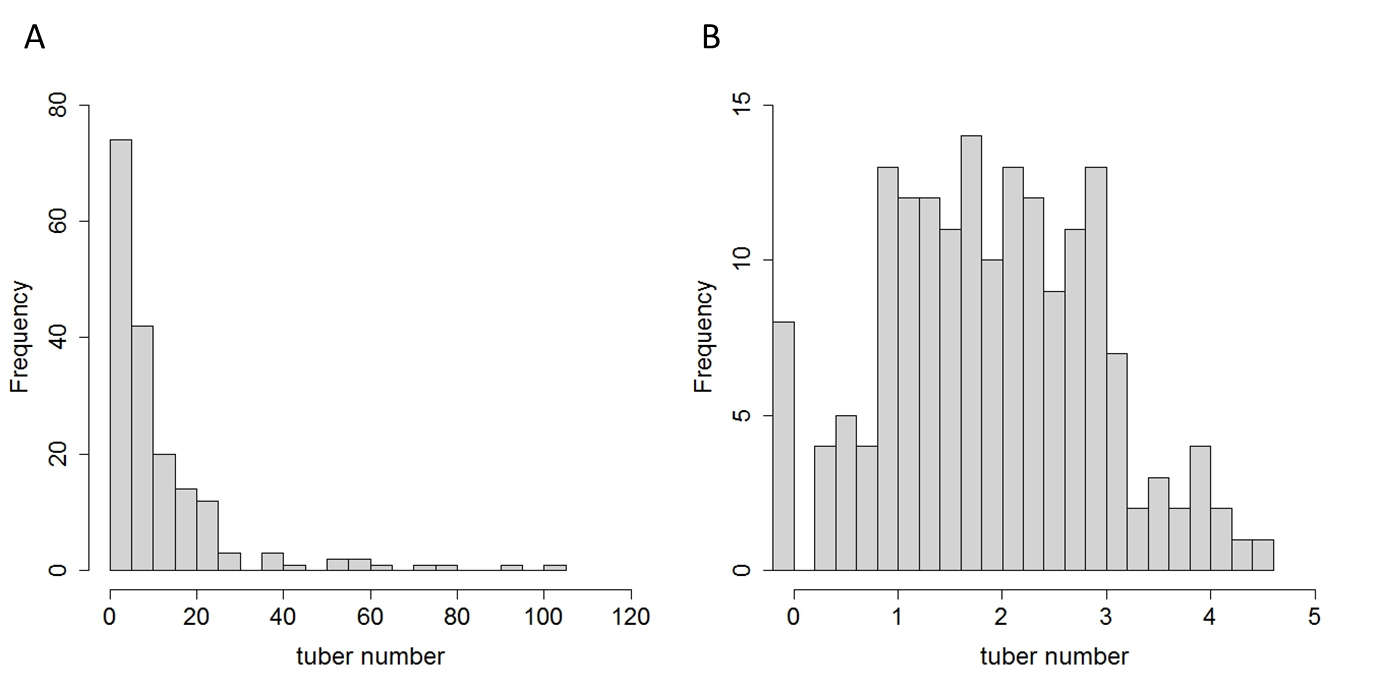


**Figure S14: Distribution of phenotypic data for average tuber number.** Distributions are shown before (A) and after (B) Box-Cox transformation of the phenotypic data.


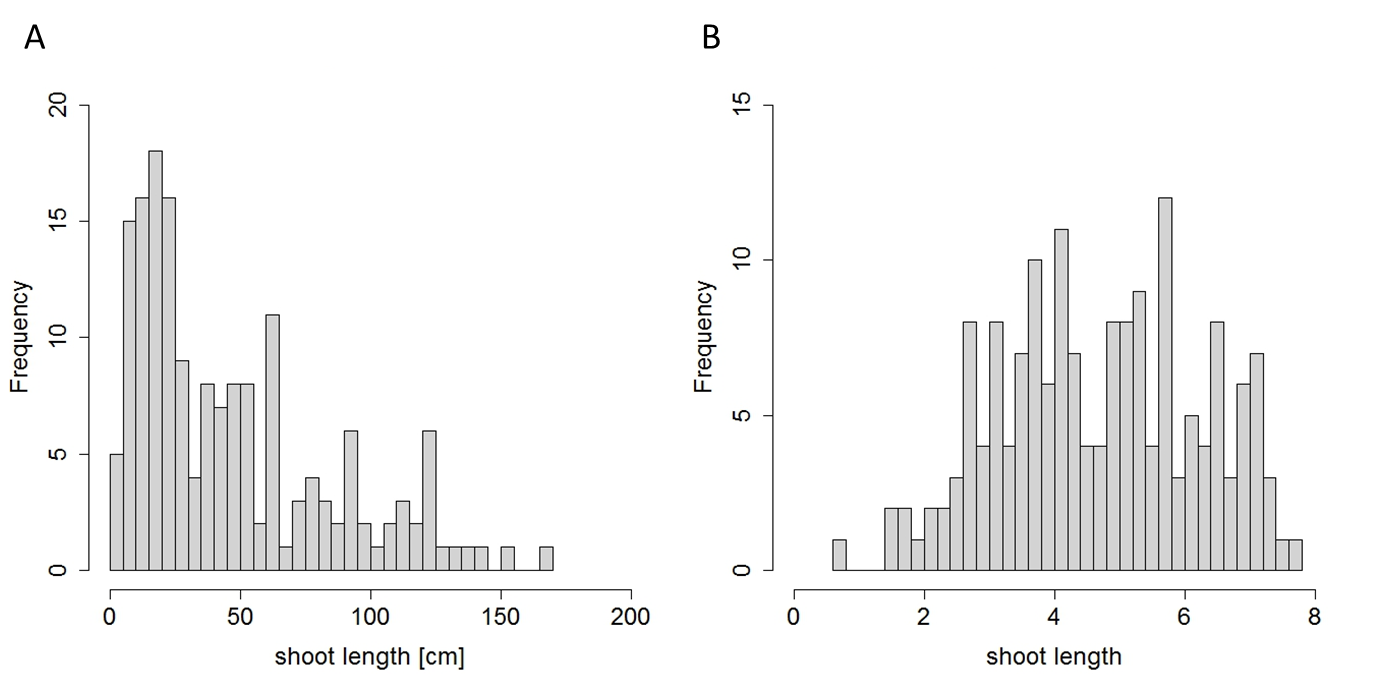


**Figure S15: Distribution of phenotypic data for average shoot length.** Distributions are shown before (A) and after (B) Box-Cox transformation of the phenotypic data.


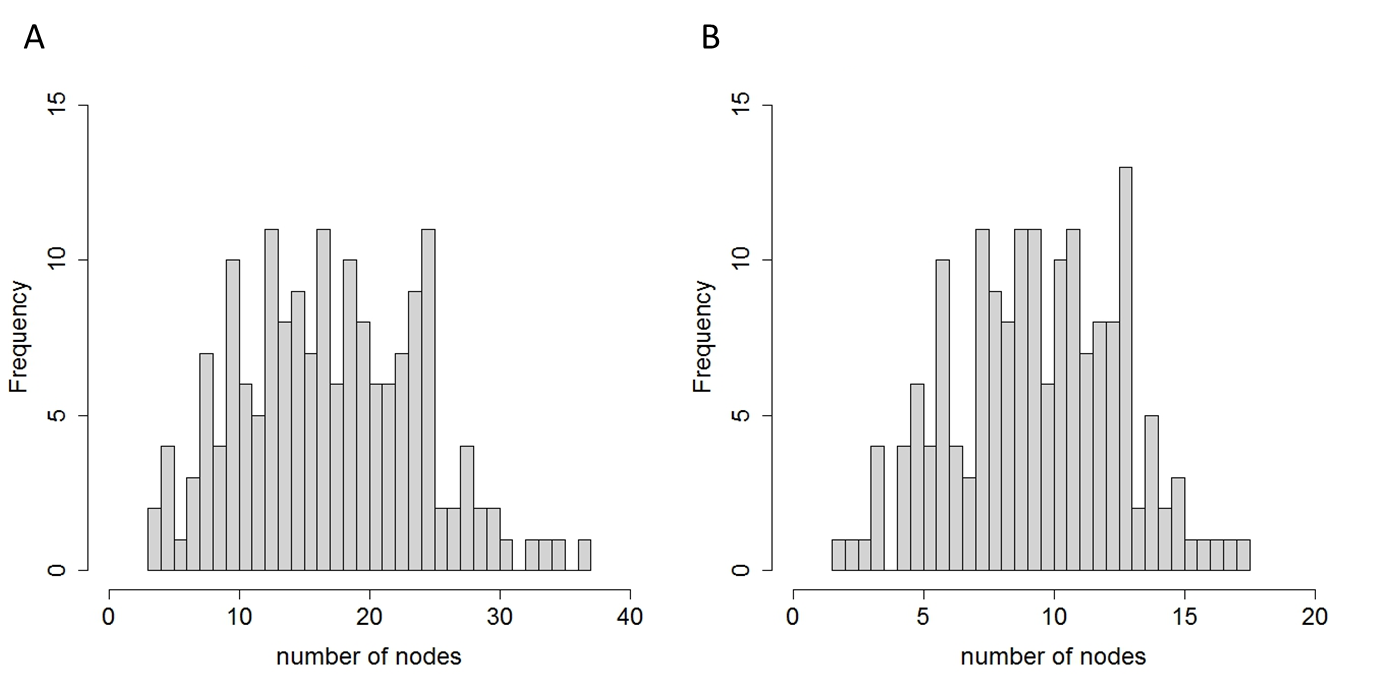


**Figure S16: Distribution of phenotypic data for average number of nodes.** Distributions are shown before (A) and after (B) Box-Cox transformation of the phenotypic data.

**
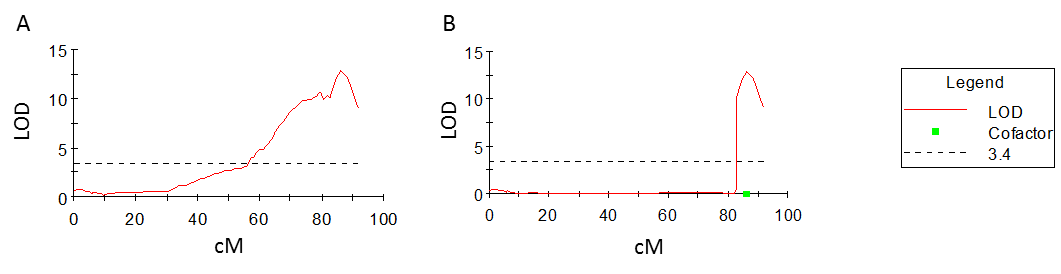
**

**Fig S17: QTL charts for Interval Mapping (A) and Multiple QTL Mapping (B) for the average tuber weight on chromosome 4.**

**
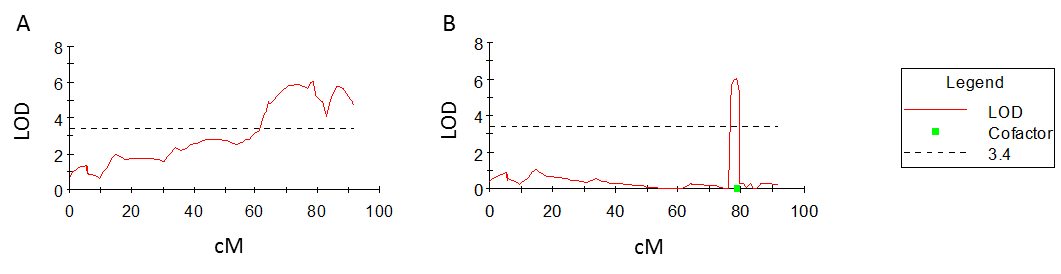
**

**Fig S18: QTL charts for Interval Mapping (A) and Multiple QTL Mapping (B) for the average tuber number on chromosome 4.**

**
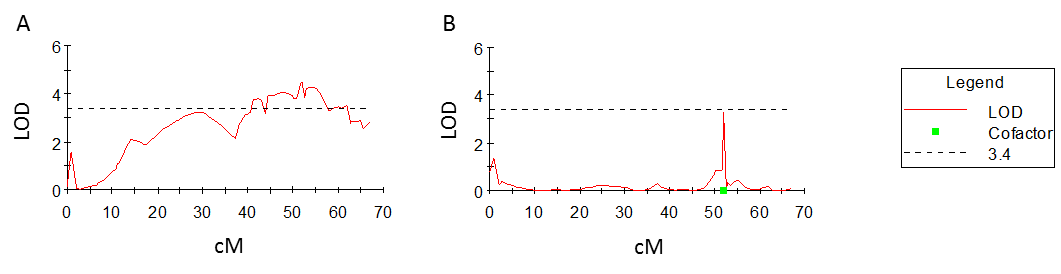
**

**Fig S19: QTL charts for Interval Mapping (A) and Multiple QTL Mapping (B) for the average shoot length on chromosome 2.**

**
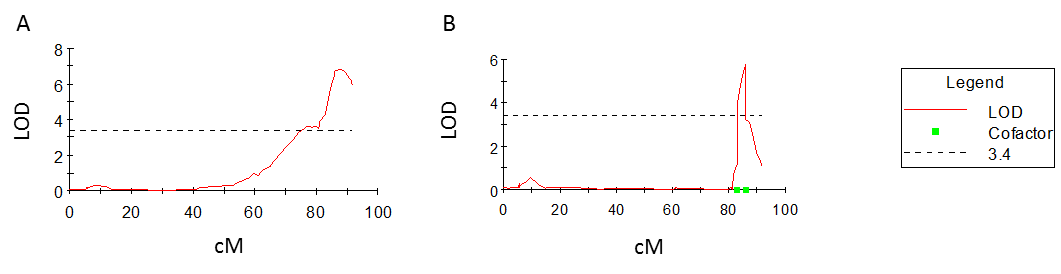
**

**Fig S20: QTL charts for Interval Mapping (A) and Multiple QTL Mapping (B) for the average shoot length on chromosome 4.**

**
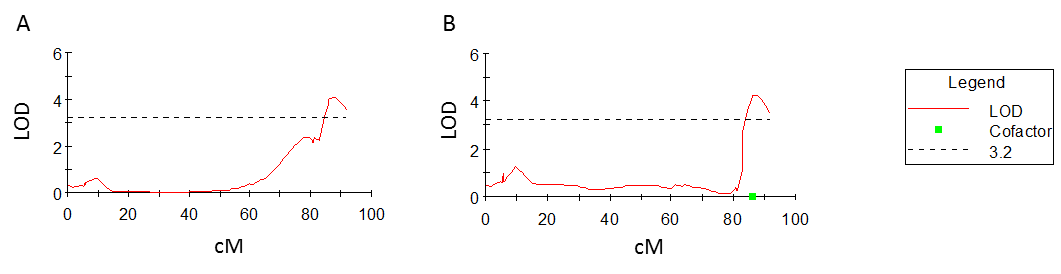
**

**Fig S21: QTL charts for Interval Mapping (A) and Multiple QTL Mapping (B) for the average number of nodes on chromosome 4.**

**
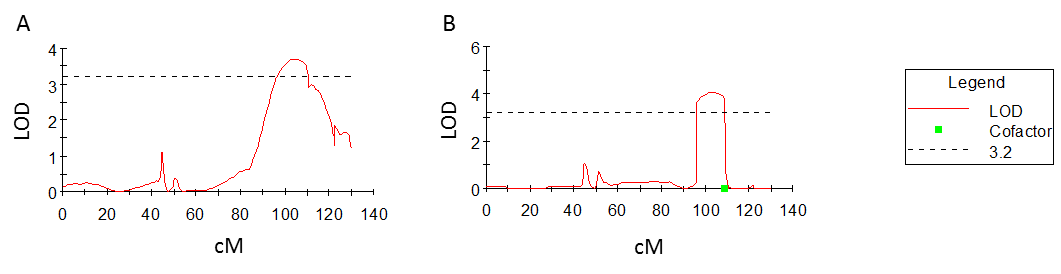
**

**Fig S22: QTL charts for Interval Mapping (A) and Multiple QTL Mapping (B) for the average number of nodes on chromosome 5.**
